# TP53 Loss Elevates NF‐κB‐IFN‐β‐MHC‐Ia Signaling to Promote NK Cell Resistance in Osteosarcoma

**DOI:** 10.1002/advs.77726

**Published:** 2026-09-11

**Authors:** Guihui Qin, Cheung Kwan Yeung, Ye Yi, Dejin Zheng, Miaoman Ye, Chi‐Chong Chio, Jinjie Wu, Siyi Fu, Chu‐Xia Deng, Ren‐He Xu

**Affiliations:** ^1^ Ministry of Education Frontiers Science Center for Precision Oncology Faculty of Medicine University of Macau Ave. de Universidade Macao SAR 999078 China; ^2^ Department of Biomedical Sciences, Faculty of Medicine University of Macau Ave. de Universidade Macau China; ^3^ Faculty of Synthetic Biology Shenzhen University of Advanced Technology Shenzhen China

**Keywords:** major histocompatibility complex, natural killer cells, osteosarcoma, p53

## Abstract

*TP53* inactivation is a key event in osteosarcoma (OS) development and underlies its aggressiveness, yet its role in tumor‐immune interactions remains poorly understood. Here, we investigate how p53 loss alters osteosarcoma susceptibility to natural killer (NK) cell‐mediated cytotoxicity using OS cell lines and stem cell‐derived OS‐associated models. We found that *TP53* loss in human osteosarcoma cell lines dysregulates NK cell regulatory ligands, specifically upregulating MHC‐Ia to confer resistance to NK cell killing. Single‐cell RNA sequencing of clinical specimens reveals mesenchymal stem cells (MSCs) is associated with OS development. Using human embryonic stem cell (hESC)‐derived MSCs, the study shows that *TP53* loss also drives MHC‐Ia overexpression and NK cell resistance via activation of the cytosolic dsDNA‐NF‐κB‐IFN‐β axis. Syngeneic mouse models confirmed that p53 loss results in more aggressive tumors with reduced NK cell infiltration than wild‐type controls. Clinically, impaired p53 function correlates with elevated MHC‐Ia expression and type I IFN signaling. These findings uncover that a *TP53* loss‐triggered NF‐κB‐IFN‐β‐MHC‐Ia axis contributes to NK cell resistance in OS development and highlight its potential as a critical therapeutic target for early intervention.

## Graphical abstract

*TP53* loss in a transforming or osteosarcoma cell promotes cytosolic DNA accumulation, activating NF‐κB‐dependent IFN‐β production. Autocrine IFN‐β signaling increases cell‐surface HLA‐Ia expression, strengthens inhibitory KIR signaling in natural killer cells, and thereby enables the affected cell to evade NK cell‐mediated cytotoxicity.

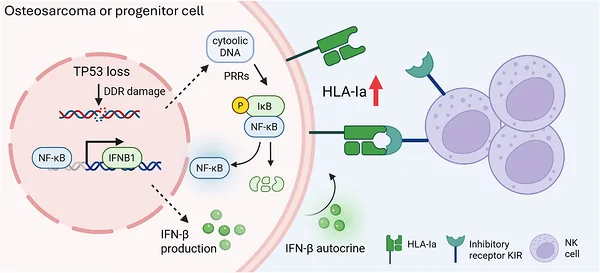

## 1 Introduction

Osteosarcoma (OS) is the most common bone cancer originating from mesenchymal cells. It is highly heterogeneous, exhibiting remarkable variations in genetic makeup, behavior, and response to treatment [1]. The tumor suppressor gene *TP53* is one of the most frequently mutated genes in diverse cancers, resulting in various statuses including wild type (WT), gain‐of‐function (GOF), dominant‐negative (DN) effect, and loss‐of‐function (LOF) mutations [2, 3]. *TP53* is frequently mutated in human OS and loss of p53 related to the sarcomagenesis was confirmed in numerous studies [4, 5, 6, 7]. Although the role of p53 and its mutant forms in tumorigenesis has been extensively studied, their roles in tumor immunity remain less understood.

During normal tissue homeostasis, newly transformed cells can emerge sporadically; however, they are often rapidly recognized and eliminated by the immune system through a process known as cancer immunosurveillance [8, 9]. Unlike adaptive tumor immunity, which requires specific signals for activation, innate immunity serves as the first barrier against malignant cell precursors, with NK cells playing a crucial role in eliminating these cells by responding to non‐specific distress signals from those experiencing homeostatic disturbances [10, 11, 12]. They patrol the body to recognize and eradicate not only malignant cells but also premalignant cells, thereby are essential in preventing tumor initiation and progression [13]. The functional status of NK cells is regulated by ligands expressed on the surface of target cells, which include NK activating and ‐inhibitory ligands [14]. NK activating ligands primarily consist of NKG2D ligands, such as *MICA/B* and *ULBP1‐6*. In contrast, NK inhibitory ligands are composed of KIR and NKG2A ligands, such as class I major histocompatibility complex (MHC‐I) molecules or human leukocyte antigens (HLA‐I). HLA‐I can be further divided into HLA‐Ia (including *HLA‐A*, *‐B*, and *‐C*) and ‐Ib (including *HLA‐E* and *‐G*).

*TP53* exerts dichotomous effects on NK cell susceptibility by differentially regulating NK ligand expression across cellular contexts [15]. On one hand, p53 activation has been reported to induce NK susceptibility in cancer cells by upregulating NK‐activating ligands such as *ULBP1* and *ULBP2* [16, 17]. On the other hand, p53 activation has also been shown to reduce NK susceptibility [18]. For instance, lung cancer and OS cell lines with WT *TP53* exhibit higher expression of the NK inhibitory ligand HLA‐Ia compared to *TP53*‐inactivated cells [19]. Furthermore, treatment with the MDM2 inhibitor nutlin‐3a, which stabilizes p53, led to MHC‐I upregulation exclusively in *TP53* WT cells [19]. These controversial results implicate that further studies are necessary to elucidate the context‐dependent role of p53 in regulating NK cell susceptibility in specific tumor type and developmental contexts. Given the role of NK cells in early immune surveillance, understanding how p53 alterations influence tumor cell sensitivity to NK cell‐mediated cytotoxicity may provide insights into immune regulation during OS development.

As a mesenchymal tumor, osteosarcoma has been modeled by mesenchymal stem cells (MSCs) both *i*n vitro and in vivo [20, 21, 22]. Human MSCs as well as osteoblasts can be derived from various sources, including somatic tissues or human pluripotent stem cells (hPSCs) [23, 24, 25]. Using induced pluripotent stem cells (iPSCs) derived from patients with Li‐Fraumeni syndrome, Lee et al. demonstrated that a *TP53* missense mutation dysregulates the imprinted gene network, leading to defects of iPSC‐derived osteoblasts and the development of OS‐associated phenotypes [26]. Despite this and many other extensive studies establishing the critical role of *TP53* alterations in OS pathogenesis [27, 28], how *TP53* loss affects NK cell susceptibility during the early stage of OS development remains unclear. In this study, we sought to address this knowledge gap by investigating how different *TP53* statuses influence NK cell susceptibility in OS cells in vitro, during OS development in vivo, and in stem cell‐derived MSC and osteogenic models associated with OS development. Emphasis was placed on how initial genetic alterations may influence tumor cell interactions with the immune system.

## 2 Results

### 2.1 P53 Loss Alters NK Regulatory Ligands and Increases NK Resistance in Human Osteosarcoma Cells In Vitro

**FIGURE 1:**
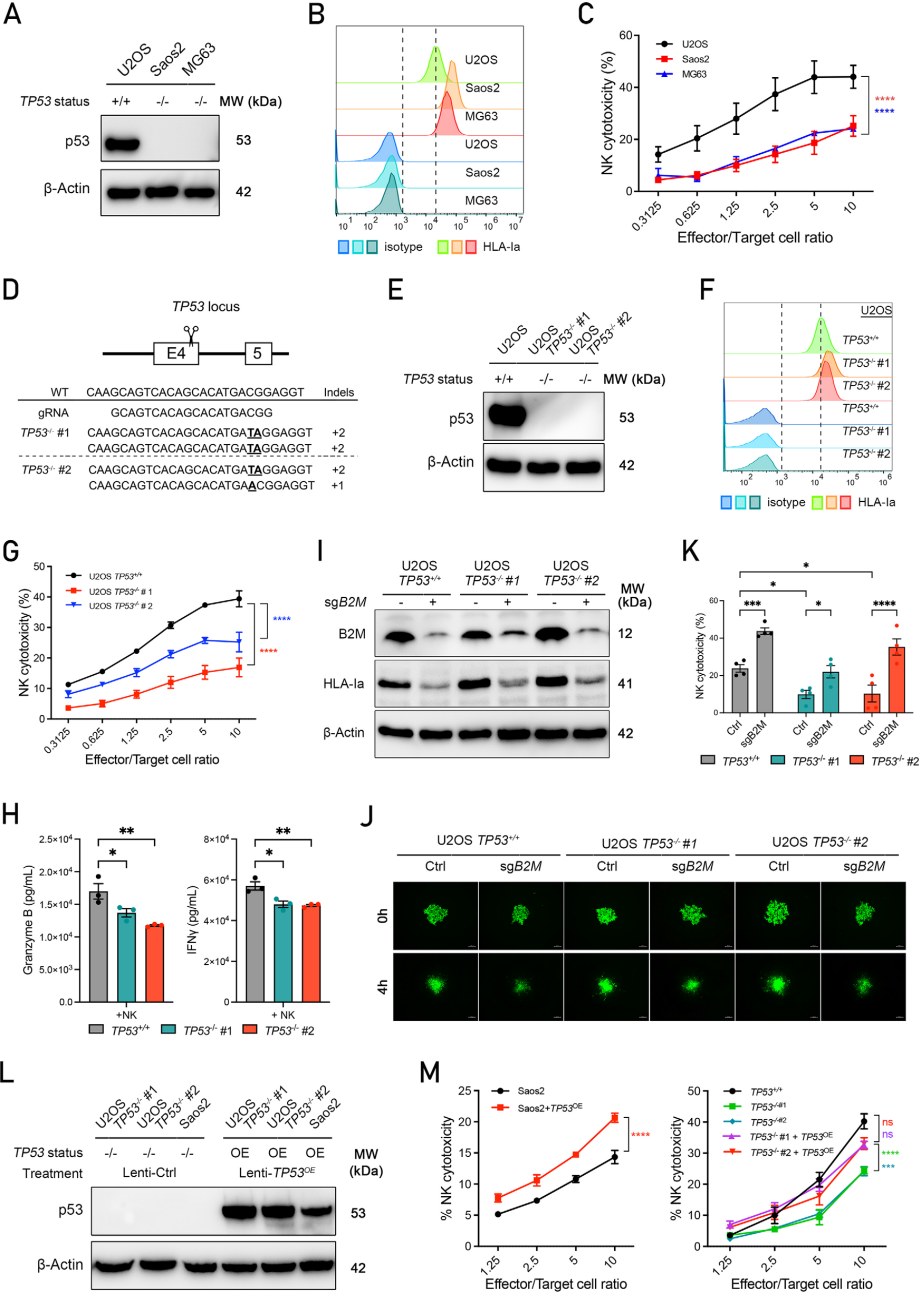
P53 loss elevates HLA‐Ia expression and NK resistance in human osteosarcoma cells. (A) Immunoblotting for p53 expression in OS cell lines. β‐actin was tested as an internal control. (B) Flow cytometry for cell‐surface HLA‐Ia expression in OS cell lines. Representative of three independent experiments (*n* = 3). (C) NK cytotoxicity assay for human primary NK cell‐mediated lysis of OS cells. Data are representative of three independent experiments (*n* = 3) as mean ± standard error of the mean (SEM), ****p < 0.0001 per ordinary two‐way ANOVA with Tukey's multiple comparisons test. Red asterisks: U2OS vs Saos2; blue asterisks: U2OS vs MG63. (D) Schematic of *TP53* knockout strategy and genotyping validation for two *TP53^−/−^* U2OS clones (#1 and #2). (E) Immunoblotting analysis of p53 expression in *TP53^+/+^* and *TP53^−/−^* U2OS cells. β‐actin was tested as an internal control. (F) Flow cytometry analysis for cell‐surface HLA‐Ia expression in OS cell lines. Representative of three independent experiments (*n* = 3). (G) NK cytotoxicity assay (refer to C). Data are shown as mean ± SEM, *n* = 3, ****p < 0.0001 per ordinary two‐way ANOVA with Tukey's multiple comparisons test. Red asterisks: *TP53^+/+^* vs *TP53^−/−^* clone #1; blue asterisks: *TP53^+/+^* vs *TP53^−/−^* clone #2. (H) ELISA for granzyme B and IFNγ secreted by primary NK cells. *n* = 3, mean ± SEM, *p < 0.05, **p < 0.01 per ordinary one‐way ANOVA followed by Dunnett's multiple comparisons test. (I) Immunoblotting for B2M and HLA‐Ia expression in OS cell lines, β‐actin was tested as an internal control. (J, K) Representative live‐cell fluorescence images showing NK cell‐mediated killing of GFP‐labeled parental and *B2M*‐knockout OS cells (J). Data are shown as mean ± SEM, *n* = 4, *p < 0.05, ***p < 0.001, ****p < 0.0001 per ordinary two‐way ANOVA with Tukey's multiple comparisons test (K). (L) Immunoblotting for p53 expression in *TP53^−/−^* and *TP53^OE^* U2OS clones and Saos2 cells, β‐actin was tested as an internal control. (M) NK cytotoxicity assay on OS cells with distinct *TP53* statuses. Data are shown as mean ± SEM, *n* = 3, ns, non‐significance. ***p < 0.001, ****p < 0.0001 per ordinary two‐way ANOVA followed by Tukey's multiple comparisons test. Right panel: red/purple ns for *TP53^+/+^* vs *TP53^OE^* U2OS clones; green/indigo asterisks for *TP53^−/−^* vs paired *TP53^OE^* clones.

To dissect the role of p53 in NK cell resistance of human OS cells, we selected three human OS cell lines with distinct *TP53* statuses, including *TP53^+/+^* U2OS, *TP53 ^−/−^* Saos2 and MG63. Their identities were confirmed via short tandem repeat analysis (Table S3), and p53 expression was verified through western blotting (Figure 1A). Consistent with previous report [29], U2OS, Saos2, and MG63 cells exhibited morphologies resembling immature osteoblasts, mature osteoblasts, and fibroblast‐like cells, respectively (Figure S1A). The *TP53^−/−^* Saos2 and MG63 cells exhibited higher cell‐surface HLA‐Ia level (Figure 1B) and greater resistance to NK cell‐mediated cytotoxicity than the *TP53^+/+^* U2OS cells (Figure 1C).

To further investigate the role of p53 in NK cell‐mediated cytotoxicity, we knocked out *TP53* in U2OS cells using CRISPR‐Cas9 targeting exon 4, one of the most common mutation sites in *TP53* [30]. *TP53* knockout (KO) was confirmed via genotyping (Figure 1D) and western blotting (Figure 1E). *TP53^−/−^* U2OS cells increased cell‐surface HLA‐Ia expression (Figure 1F) and NK cell resistance (Figure 1G). RNA‐seq analysis on the *TP53^+/+^* U2OS cells and two isogenic *TP53^−/−^* clones showed that up‐regulated genes in *TP53^−/−^* U2OS cells were predominantly enriched in virus response‐related pathways and type I interferon (IFN) signaling‐related pathways (Figure S1B). As shown in a heatmap, NK inhibitory ligands, especially the three HLA‐Ia genes and *HLA‐E*, were expressed higher in *TP53^−/−^* clones, whereas NK activating ligands including *MICA*, *ULBP2*, *ULBP3*, *NECTIN2*, and *BAG6* expressed lower than the *TP53^+/+^* controls (Figure S1C). These results indicate that p53 loss may trigger IFN responses and alter the expression of the NK‐regulatory ligands.

Consistent with these ligand changes, NK cells co‐cultured with *TP53^−/−^* target cells, which expressed at higher levels of HLA‐Ia, secreted lower levels of granzyme B and IFN than those co‐cultured with *TP53^+/+^* cells (Figure 1H), suggesting reduced NK effector activation. To further assess whether HLA‐Ia molecules contributed to NK resistance, β2‐microglobulin (*B2M*) KO was performed in OS cells (Figure 1I), because B2M is required for cell‐surface expression of HLA‐Ia/MHC class I molecules. Following *B2M* KO, both *TP53^+/+^* and *TP53^−/−^* OS cells exhibited reduced HLA‐Ia and increased sensitivity to NK cell‐mediated cytotoxicity (Figure 1JK). In parallel, NK cells co‐cultured with *B2M*‐KO target cells showed increased secretion of IFN‐γ and granzyme B (Figure S1E), suggesting a partial restoration of NK cell effector function. These results support the involvement of HLA‐Ia inhibitory ligands in NK resistance of OS cells.

Conversely, lentiviral transduction for *TP53* overexpression (OE) in *TP53^−/−^* U2OS and Saos2 cells resulted in a *TP53^−/−^*‐to‐*TP53^OE^* (*TP53^OE^*) genotype (Figure 1L), reversed the expression of cell‐surface HLA‐Ia (Figure S1D), and partially reduced the NK cell resistance (Figure 1M). Together, these results suggest that p53 loss is associated with increased cell‐surface HLA‐Ia presentation and hence increases the NK cell resistance of OS cells.

### 2.2 P53 Loss Promotes NK Resistance and Tumor Establishment of Human Osteosarcoma Cells In Vivo

**FIGURE 2:**
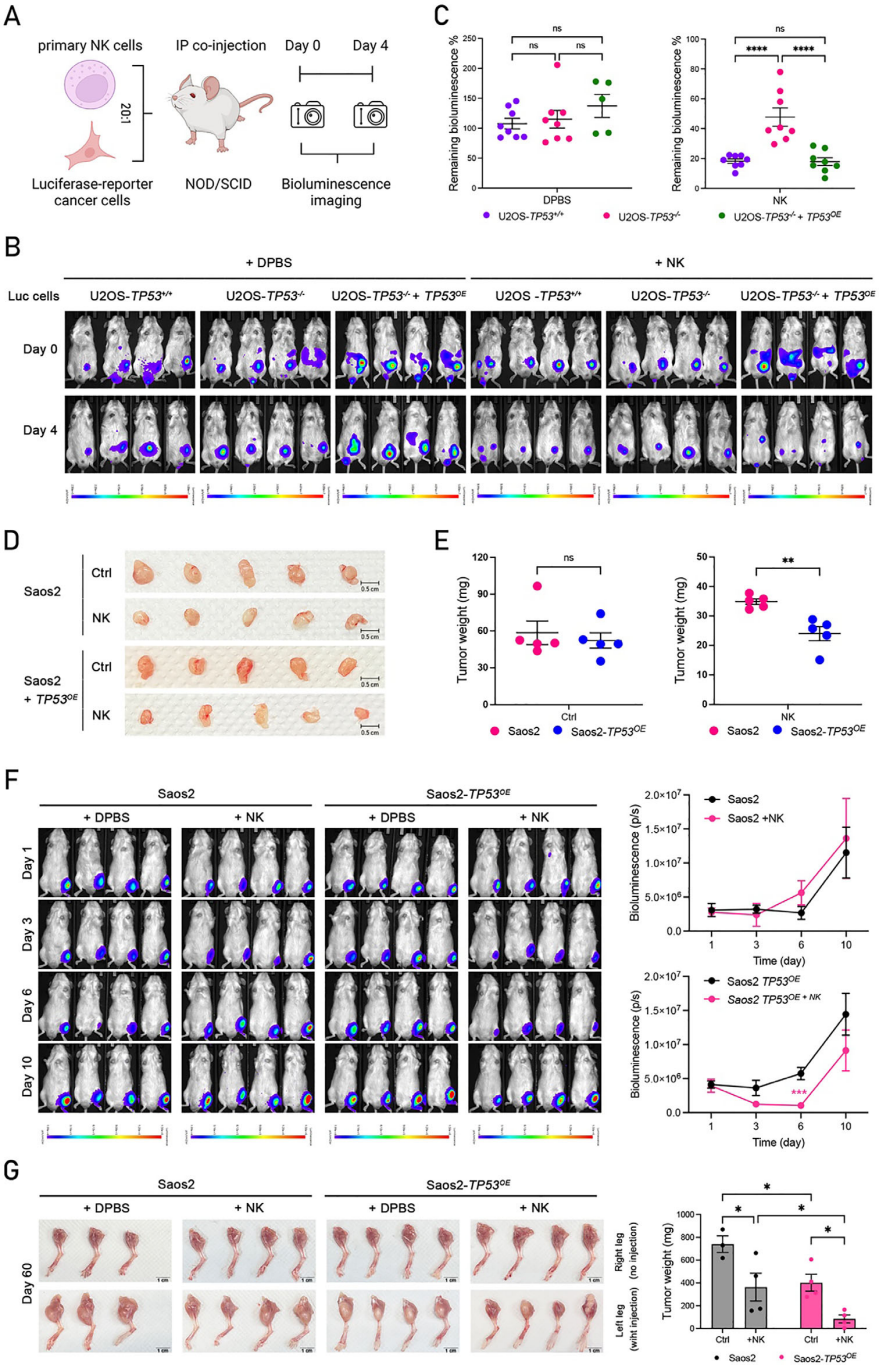
P53 loss increases NK resistance and tumorigenesis of human osteosarcoma cells in vivo. (A) Schematic workflow for NK cell‐mediated cytotoxicity against luciferase (Luc) expressing OS formed in NOD/SCID mice. (B, C) Representative images (B) and dot plots (C) for remaining bioluminescence in the mice 4 days after co‐injection of primary NK cells (or an equivalent amount of DPBS) and the Luc^+^ *TP53^+/+^*, *TP53^−/−^* and *TP53^OE^* U2OS cell lines at a cell ratio of 20:1. Data are shown as mean ± SEM, *n* = 5–8. ns: non‐significance. ****p < 0.0001 per ordinary one‐way ANOVA followed by Tukey's multiple comparisons test. (D, E) Subcutaneous xenografts 3 weeks after the co‐injection of primary NK cells (or DPBS) and Luc^+^ Saos2 cells at a cell ratio of 5:1. Displayed are representative images of the tumors (D) and dot plots for their weight (E). Scale bar, 0.5 cm in D. Data are shown as mean ± SEM in E. *n* = 5. ns: non‐significance, **p < 0.01 per unpaired Student's *t* test. (F) Representative images (left) and dot plots (right) for remaining bioluminescence in NSG mice at days 1, 3, 6, and 10 after intratibial injection of Luc^+^ Saos2 cells. Data are shown as mean ± SEM, *n* = 4. *p < 0.05 per unpaired *t* test. (G) Xenografts in NSG mice at day 60 after intratibial injection of Saos2 OS cells. Displayed are representative images of the tumors (left) and dot plots for their weight (right). Data are shown as mean ± SEM, *n* = 3–4. *p < 0.05 per ordinary two‐way ANOVA following multiple comparisons.

To validate the p53‐regulated NK cell resistance in vivo, we constructed *TP53^+/+^*, *TP53^−/−^*, and *TP53^OE^* U2OS OS cell lines with stable expression of luciferase through lentiviral transduction. Luciferase transduction did not alter p53 statuses (Figure S2A) and cell‐surface expression of HLA‐Ia (Figure S2B). Based on a previously reported approach [31], luciferase‐expressing OS cells were then co‐injected intraperitoneally with primary human NK cells at a ratio of 20:1 into NOD/SCID mice, while control mice received tumor cells mixed with an equal volume of DPBS. Bioluminescent signals were measured on days 0 and 4 post‐injection (Figure 2A), and the percentage of remaining bioluminescence was used to assess in vivo NK cell resistance (Figure 2B,C). No obvious difference was observed among DPBS treatment groups at day 4 (Figure 2B,C, left). In contrast, among NK cell treatment group, the *TP53^−/−^* tumor‐bearing mice retained higher bioluminescent signals than the *TP53^+/+^* tumor‐bearing mice at day 4, and such difference was no longer observed in mice bearing *TP53^OE^* tumors (Figure 2B,C, right). These results suggest that p53 loss is responsible for the initial clearance of U2OS OS cells by NK cells in vivo.

To test the role of p53 loss in establishment of OS, *TP53^−/−^* or *TP53^OE^* Saos2 cells were subcutaneously co‐injected with or without human primary NK cells into NOD/SCD mice at a tumor cell to NK cell ratio of 1:5. At day 21 post‐injection, tumors formed in situ were resected, photographed (Figure 2D), and weighed (Figure 2E). In the presence of NK cells, tumors were larger and heavier in the *TP53^−/−^* group than the *TP53^OE^* group, consistent with the bioluminescence‐retention results observed above at the initial stage. To address the concern that OS generated by subcutaneous inoculation of the tumor cells did not arise in an orthotopic environment, an intratibial OS model was employed in NSG mice [32]. Only Saos2, but not U2OS, cells exhibited obvious tumor‐forming capacity in this model (Figure S2C). Consistent with the results from the subcutaneous model, *TP53^OE^* Saos2 cells formed orthotopic tumors smaller than those formed by Saos2 cells (which lacked p53 functions) (Figure 2F,G). Together, these in vivo data suggest that p53 loss contribute to the resistance of OS cells to NK cell‐mediated cytotoxicity in vivo, thereby promoting tumor survival and establishment.

Members of the p53 family exhibit both overlapping and distinct functions, and p53 loss can induce TAp73 expression in certain contexts through E2F1‐mediated transcriptional regulation [33]. However, genetic studies indicate that p63 and p73 do not universally compensate for p53 loss, as p53 also exerts tumor‐suppressive functions independently of its homologs [34, 35]. Consistent with this context‐dependent relationship, we observed p73 upregulation in *TP53^−/−^* OS cell lines but not consistently in *TP53^−/−^* ESCs or MSCs, while p63 expression remained largely unchanged (Figures S2D and S6H). These findings suggest that p73 upregulation is cell type‐dependent and unlikely to represent a common mechanism underlying the p53 loss‐associated immune phenotype across our models.

### 2.3 P53 Loss Promotes Mouse Osteosarcoma with Reduced NK Cell Infiltration in Syngeneic Mice

**FIGURE 3:**
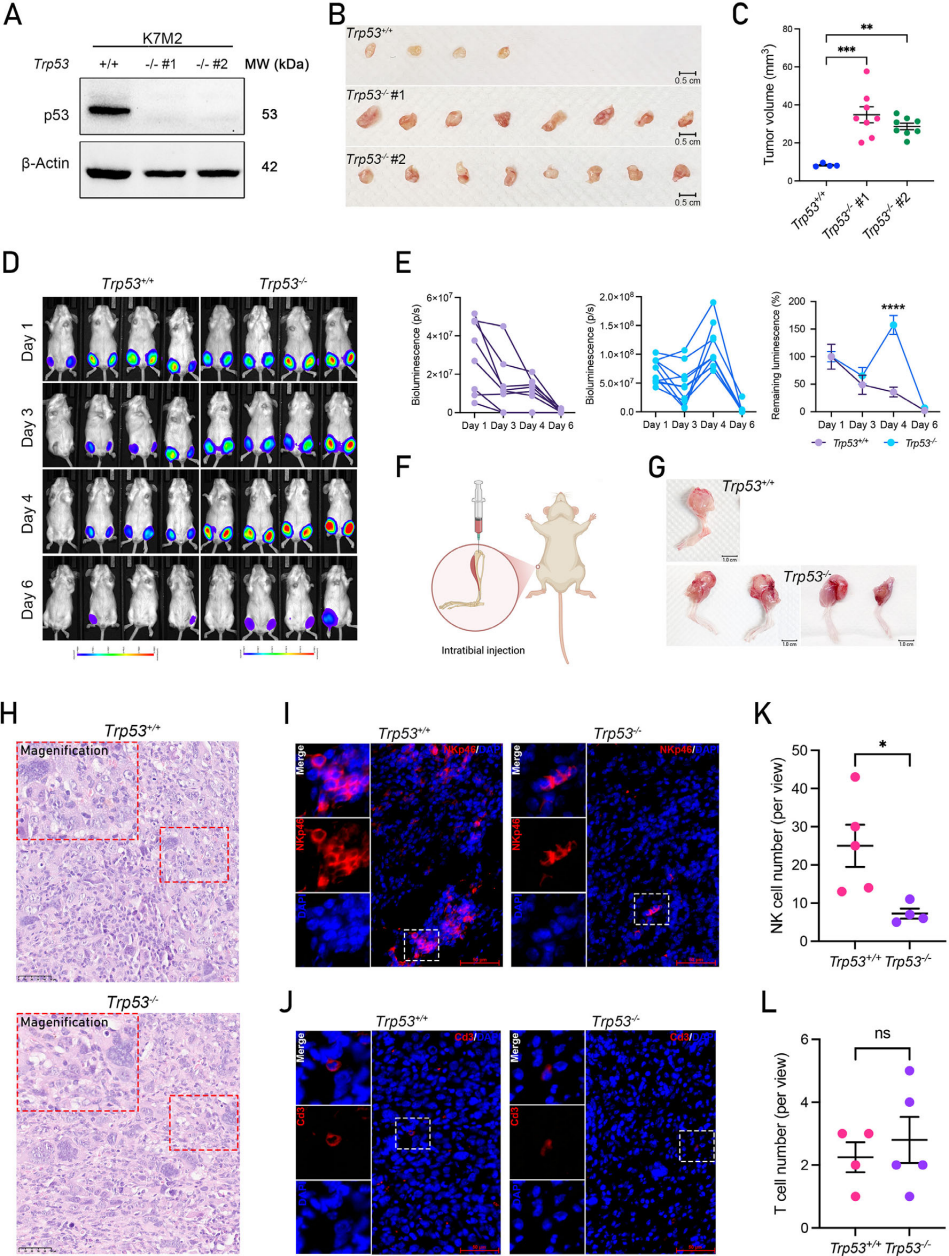
p53 loss promotes mouse osteosarcoma development in syngeneic mice, associated with reduced NK cell infiltration. (A) Immunoblotting for expression of p53 in the *Trp53^+/+^* mouse K7M2 OS cells and two *Trp53^−/−^* subclones derived via *Trp53* KO of K7M2 cells. β‐actin was tested as an internal control. (B) Images of allografts excised at day 7 after subcutaneous injection of the OS cells into both thighs of BALB/c mice. Scale bar, 0.5 cm. *n* = 4. (C) Dot plot for the tumor volume with data shown as mean ± SEM. *n* = 4–8. **p < 0.01, ***p < 0.001 per ordinary one‐way ANOVA followed by Dunnett's multiple comparisons test. (D, E) Representative images (D) and line charts (E) for remaining bioluminescence of luciferase‐expressing OS cells at various days after intramuscular injection into both thighs of BALB/c mice. Mouse number = 4. (F‐G) Schematic of an orthotopic model via intratibial injection (F) and pictures of orthotopic tumors (G). (H) Representative images of H&E‐stained sections of *Trp53^+/+^* and *Trp53^−/−^* K7M2 tumors. Scale bar, 50  µm. (I‐J) Immunofluorescence analysis for expression of the NK cell marker Nkp46 (I) and T cell marker CD3ε (J) in the *Trp53^+/+^* and *Trp53^−/−^* tumors. (K‐L) Dot plots for infiltration of NK (K) or T (L) cells in tumors. Data are shown as mean ± SEM, *n* = 4–5. ns, non‐significance, *p < 0.05 per unpaired *t* test. Scale bar, 50 µm.

To further validate these findings in an immunocompetent setting, we established a syngeneic mouse model using K7M2, a murine OS cell line [36] inoculated into BALB/c mice. As K7M2 cells expressed p53, *Trp53* KO clones were generated using CRISPR‐Cas9. Successful KO was confirmed in two independent clones (Figure 3A). Both WT (*Trp53^+/+^*) and KO (*Trp53^−/−^*) K7M2 clones were genetically engineered to express luciferase via lentiviral transduction. According to the original report describing the K7M2 OS model, K7M2 cells can be used to establish tumors by subcutaneous injection, direct orthotopic injection, or intramuscular implantation followed by transplantation of the resulting tumor fragments into an orthotopic site [37]. Both WT and KO K7M2 cells were injected subcutaneously into BALB/c mice followed by tumor excision on day 7 from the injection sites. Tumors derived from *Trp53^−/−^* cells were significantly larger in volume compared to those from *Trp53^+/+^* cells (Figure 3B,C), indicating that p53 loss accelerates tumor growth in this model. Similar results were observed in the intramuscular model, in which *Trp53^+/+^* cells exhibited lower tumor persistence than *Trp53^−/−^* cells reflected by remaining bioluminescence signals in the injection sites (Figure 3D,E).

In the orthotopic intratibial model, *Trp53^−/−^* K7M2 cells also exhibited a higher rate (4/12 vs 1/12) of tumor formation than *Trp53^+/+^* cells in the injection sites (Figure 3F,G). Histological analysis confirmed that tumors in both groups displayed typical OS features in the intratibial (Figure 3H) as well as intramuscular (Figure S2F) models. Consequently, reduced NK cell infiltration was found in *Trp53^−/−^* tumors compared to *Trp53^+/+^* tumors (Figure 3I–K). In addition, T cell infiltration was low across all tumors without significant differences between the *Trp53^+/+^* and *Trp53^−/−^* groups (Figure 3J–L). These findings suggest that p53 loss also promotes OS genesis in the syngeneic model, which is associated with diminished NK cell infiltration.

### 2.4 scRNA‐Seq Suggests an MSC‐Associated Cellular Origin of Osteosarcoma and Its Crosstalk with NK Cells

**FIGURE 4:**
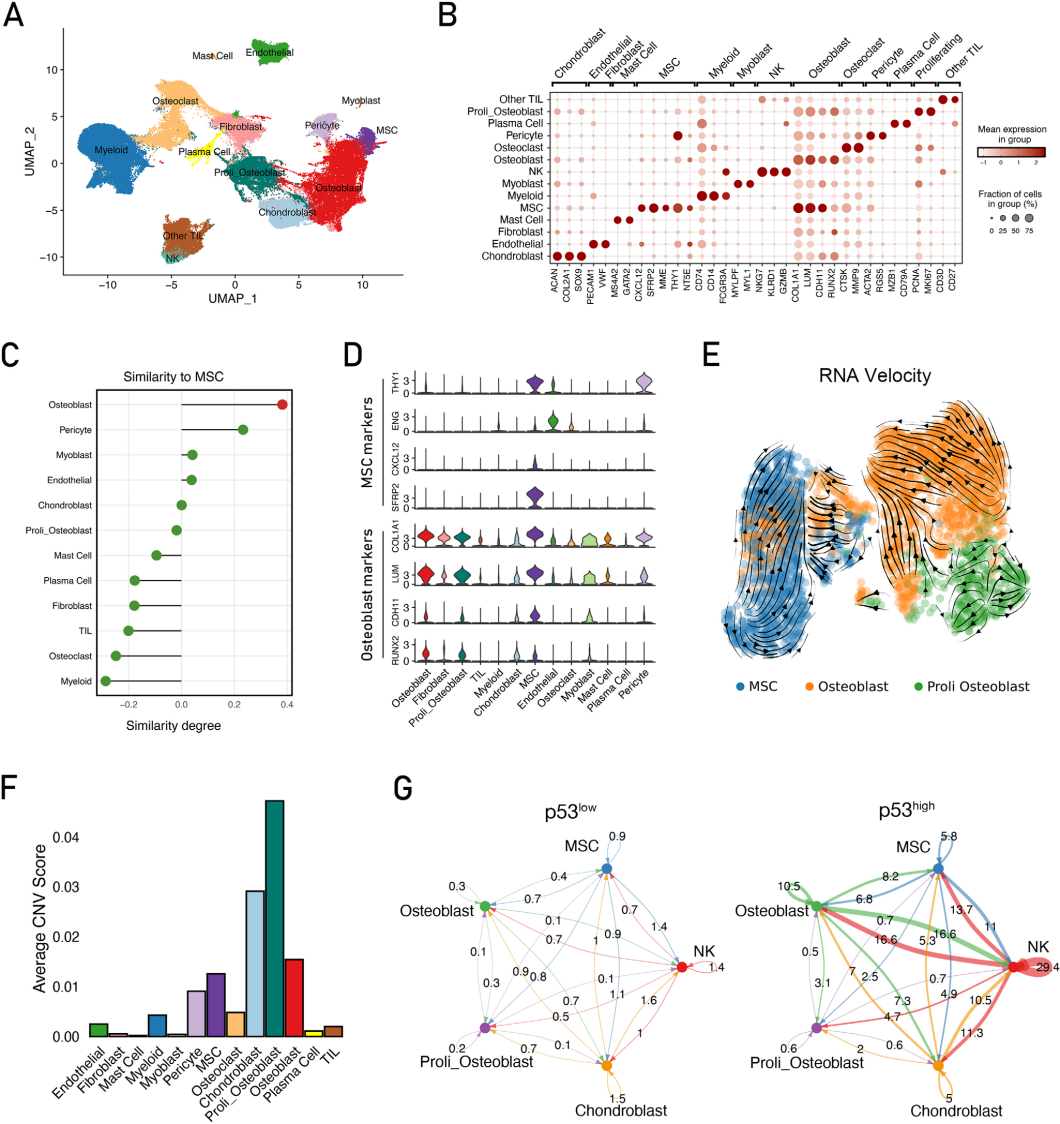
scRNA‐seq reveals MSC‐originated osteosarcoma progression and crosstalk with NK cells. (A) UMAP dimensionality reduction for major cell type annotation of all single cells in OS samples. (B) Multi‐group dot plot for gene expression signatures (columns) across the major cell types (rows). (C) Evaluation of similarity to MSCs for the major cell types. (D) Violin plots for levels of osteoblast and MSC markers across the major cell types. (E) RNA velocity streamlines for pseudotime differentiation directions of MSCs, proliferating osteoblasts, and osteoblasts. (F) InferCNV analysis showing genome‐wide CNV burden across the major cell types. (G) Circle plot for interaction weights among some of the major cell types based on their p53 function levels which were divided into p53^low^ and p53^high^.

The above results prompted us to look for comprehensive understanding of the cellular landscape in an OS, we analyzed scRNA‐seq datasets GSE152048 and GSE162454 which contained OS samples from 17 patients [38, 39]. After removing the low‐quality cells that exhibited fewer than 200 detected genes or had over 10% mitochondria‐associated genes, 145,197 cells were filtered for unbiased clustering. According to their gene expression profiles and canonical markers, 14 main clusters were identified through the uniform manifold approximation and projection (UMAP) analysis (Figure 4A): (1) plasma cells: *MZB1* and *CD79A*; (2) pericytes: *ACTA2* and *RGS5*; (3) osteoclasts: *CTSK* and *MMP9*; (4) osteoblasts: *COL1A1*, *CDH11*, and *RUNX2*; (5) proliferating osteoblasts: *COL1A1*, *CDH11*, *RUNX2*, *PCNA*, and *MKI67*; (6) tumor infiltrating lymphocytes (TILs): *CD3D* and *CD27*; (7) NK cells: *NKG7*, *KLRD1*, and *GZMB*; (8) myoblasts: *MYLPF* and *MYL1*; (9) myeloid cells, *CD74*, *CD14*, and *FCGR3A*; (10) MSCs: *CXCL12*, *SFRP2*, *MME*, *THY1*, *NT5E*, *CD44*, and *ENG*; (11) Mast cells: *NS4A2* and *GATA2*; (12) fibroblast: *COL1A1*; (13) endothelial cells: *VWF*, *PECAM1*; (14) chondroblasts: *COL2A1*, *SOX9*, and *ACAN*. These cell type proportions among each sample are shown in Figure S3A. The overall gene profile of the 14 major cell clusters is visualized in a dot plot with annotated canonical markers indicated alongside (Figure 4B).

MSCs can typically differentiate into osteoblast, chondroblast, and adipocytes in vitro [25, 40]. Based on that, we conducted a similarity evaluation, which suggests the highest similarity of osteoblasts to MSCs (Figure 4C). In addition, MSCs expressed not only the canonical in vitro MSC markers *THY1* (CD90), *NT5E* (CD73), *CD44*, and *ENG* (CD105), but also the osteoblast markers *COL1A1*, *LUM*, *CDH11*, *RUNX2* (Figure 4D). Moreover, the developmental potential calculated by CytoTRACE2 [41] reveals that MSCs and the malignant components of OS, specifically osteoblasts and chondroblasts, are situated in a low differentiation stage characterized by a potency score exceeding 0.4 (Figure S3B). These observations imply a shared molecular signature and similar developmental potential between MSCs and osteoblasts. Finally, we applied scVelo to evaluate the dynamic cell fate of MSCs and osteoblasts through their RNA velocity [42]. MSCs and osteoblasts exhibited similar RNA proportions (Figure S3C) and the velocity streamlines corresponded to the osteoblast lineage, in which both proliferating osteoblasts and MSCs developed into osteoblasts (Figure 4E).

It has been widely recognized that the copy number variation (CNV) burden is associated with tumorigenesis. High CNV burden correlates with tumor metastasis and resistance to therapy [43], thus single‐cell CNV analysis is used to distinguish malignant cells from normal cells [44, 45]. By using inferCNV as an analyzing software and endothelial cells as a normal cell control, we evaluated the CNV burden in the major cell types in the OS samples (Figure S3D). As expected, the malignant components of OS, including osteoblasts and chondroblasts, exhibited high CNV burden. Interestingly, some traditionally assumed non‐malignant cells, especially MSCs, also displayed a noticeable degree of CNV burden (Figure 4F). These observations indicate that the MSCs with increased CNV burden may represent an early state associated to OS development.

To elucidate the association between p53 function and immune cell communications within OS samples, we evaluated the p53 activity in individual cells using UCell analysis [46] and found various p53 function levels among the cells (Figure S3E). Based on that, the samples were classified into two groups: p53^low^ and p53^high^. Then, CellChat was employed to infer the interaction scores among the major cell types within the p53^low^ and p53^high^ groups based on transcriptional patterns of ligand–receptor (L‐R) pairs from its curated database. The overall communication weight represents the aggregated strength of significant inferred L‐R interactions between cell populations. Based on this metric, cells in the p53^high^ environment exhibited stronger inferred communications with immune cells, especially with NK cells, than cells in the p53^low^ environment (Figure S3F; Figure 4G, and Tables S5 and S6). These data suggest that p53 function is associated with differential communication patterns between OS‐associated cell populations and NK cells, potentially influencing NK cell‐mediated immune interaction in OS.

### 2.5 Generation and Characterization of *TP53^−/−^* hESC‐Derived MSCs for Osteosarcoma Modeling

**FIGURE 5:**
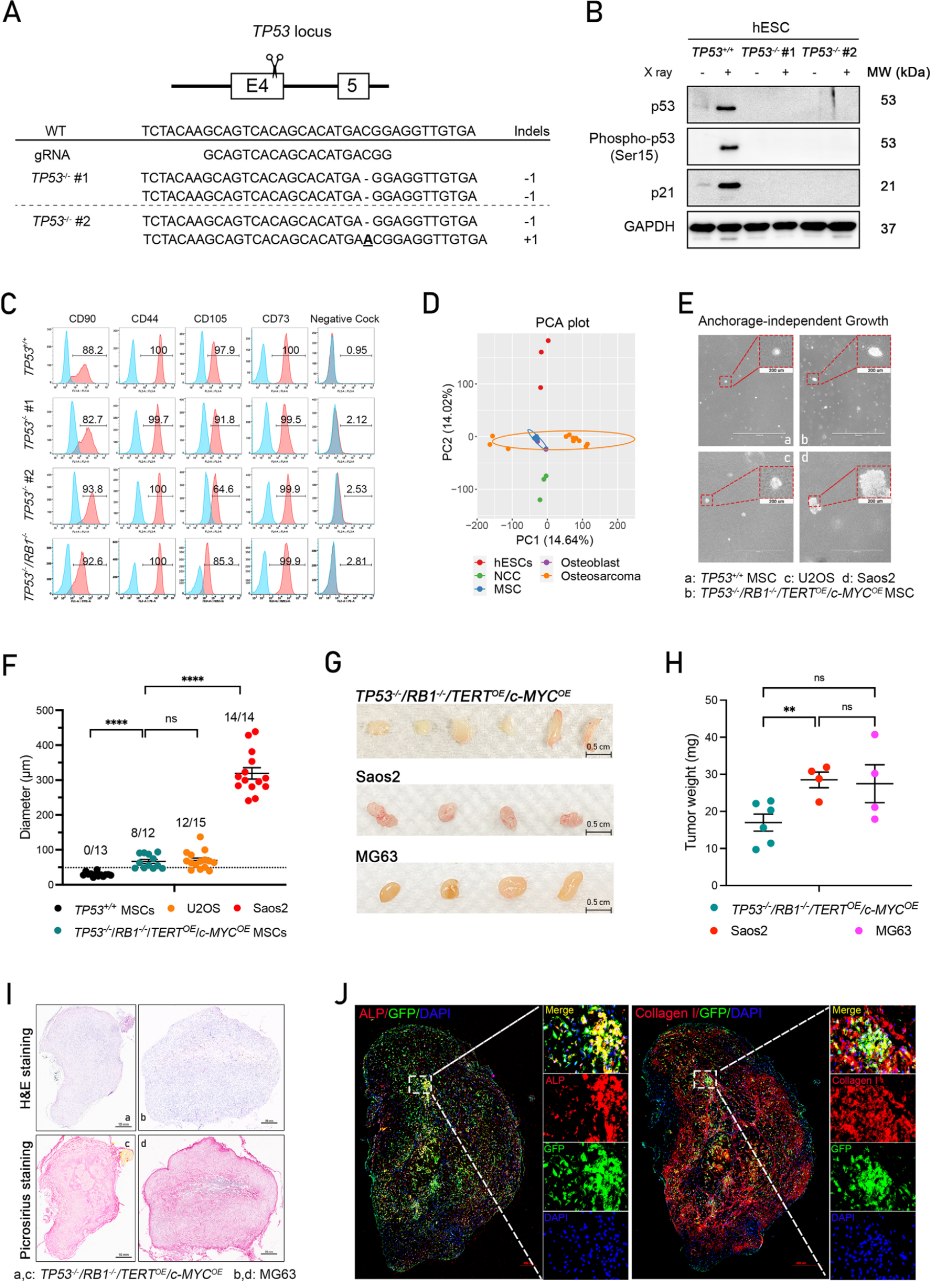
Generation and characterization of *TP53^−/−^* hESC‐derived MSCs for osteosarcoma modeling. (A) Schematic of *TP53* knockout strategy and genotyping validation for two *TP53^−/−^* hESC clones. (B) Immunoblotting analysis for expression of p53, phosphorylated p53, and p21 in hESCs. GAPDH was tested as an internal control. (C) Flow cytometry analysis for MSC markers CD90, CD44, CD105, and CD73 as well as a cocktail of negative markers in hESC‐derived MSCs. (D) PCA plot of RNA‐seq profiles for hESCs (red), NCCs (green), MSCs (blue), MSC‐derived osteoblasts (purple), and OS cell lines (orange). (E) AIG assay showing colonies formed by designated cells at day 33 post‐seeding. Scale bar, 200 µm. (F) Dot plot for the number and size of the colonies in E. Only colonies with the diameter over 50 µm were counted. Data are shown as mean ± SEM, *n* = 12–15. ns, non‐significance. ****p < 0.0001 per unpaired t test. (G) Image of subcutaneous xenografts dissected at day 20 post‐injection of designated cells. Scale bar, 0.5 cm. (H) Dot plot for the weight of xenografts in G. Data are shown as mean ± SEM, *n* = 4–6. Ns, non‐significance. **p < 0.01 per ordinary one‐way ANOVA followed by Tukey's multiple comparisons test. (I) H&E and picrosirius staining of a tumor formed at day 20 after subcutaneous injection of *TP53^−/−^*/*RB1^−/−^*/*TERT^OE^*/*c‐MYC^OE^* MSC‐derived osteocytes. A tumor formed by MG63 OS cells was stained as a control. Scale bar, 10 mm for a—c, 20 mm for b—d. (J) Immunofluorescence analysis for ALP and collagen I expression in a tumor formed by *TP53^−/−^*/*RB1^−/−^*/*TERT^OE^*/*c‐MYC^OE^* MSC‐derived osteocytes which were also positive for GFP co‐expressed from the c‐MYC‐expressing vector. Scale bar, 200 µm.

The pluripotency of hESCs allows them to differentiate into various somatic cell types, including MSCs, which makes them a powerful tool for genetic editing and disease modeling [24, 47]. Based on above results, we knocked out *TP53* in hESCs and then differentiated the cells into MSCs to establish a model for early cellular events associated with OS development. Using the strategy applied for *TP53* KO in the human OS cell lines, two *TP53^−/−^* clones #1 and #2 in the H9 hESC line were generated [48]. Genotyping (Figure 5A) and western blotting (Figure 5B) confirmed the KO of *TP53* and the loss of p53 including its total protein and phosphorylated form as well as its downstream effector p21 in *TP53^−/−^* hESCs following 8‐Gy X‐ray irradiation. It is worth noting that p53 is normally maintained at a very low level in hESCs due to its continuous degradation by MDM2‐mediated ubiquitination [49, 50]. DNA damage, such as that caused by irradiation, triggers p53 phosphorylation, which then upregulates the expression of the cyclin‐dependent kinase inhibitor p21 (WAF1/CIP1), leading to cell cycle arrest at the G1 phase [51]. No off‐target effect was observed based on genotyping of the top five predicted off‐targets for the *TP53*‐specific sgRNA (Figure S4A,B) and the whole genome sequencing (Table S4). However, karyotyping shows aneuploidies that developed in the two *TP53^−/−^* hESC clones (Figure S5A), indicating genetic instability caused by the *TP53* KO. Since p53 promotes the differentiation of pluripotent cells to maintain their genomic stability [52, 53], we tested whether p53 loss affects the pluripotency of hESCs. Through immunofluorescence staining (Figure S5B) and flow cytometry (Figure S5C), the expression of pluripotency markers NANOG, OCT4, and TRA‐1‐60 in *TP53^−/−^* hESCs was confirmed. Furthermore, after inoculation into NOD/SCID mice, *TP53^−/−^* hESCs formed teratomas, which appeared larger than those formed from *TP53^+/+^* hESCs (Figure S5D), yet both included cell types from all the three germ layers, i.e., the ectoderm, mesoderm, and endoderm (Figure S5E). Thus, the established *TP53^−/−^* hESCs lines were confirmed to possess sustained pluripotency and without noticeable off‐target effects.

The loss of p53 alone is often insufficient to initiate tumorigenesis. Additional genetic alterations (also called subsequent hits), such as the inactivation of other tumor suppressor genes like *PTEN* and *RB1*, and the overexpression of oncogenes like *TERT*, *HRAS^G12V^*, and *c‐MYC*, are often required [54]. Thus, we knocked out *RB1* in *TP53^−/−^* hESCs via CRISPR‐Cas9, which was confirmed through genotyping (Figure S6A). Next, both *TP53^+/+^* and *TP53^−/−^* hESCs were differentiated into MSCs through an intermediate stage of neural crest cells (NCCs) (Figure S6B) as reported [55]. The expression of markers for NCCs (Figure S6C,D) and MSCs (Figure 5C) was confirmed in the intermediate and subsequent cell types, respectively. All the WT and KO MSCs could further differentiate into osteoblasts, adipocytes, and chondrocytes (Figure S6E), indicating the sustained multipotency in the KO MSCs. Then, *TERT* and c‐*MYC* were overexpressed in the *RB1^−/−^*/*TP53^−/−^* MSCs through lentiviral transduction (Figure S6F) to increase the tumorigenicity of the cells.

To assess the OS modeling potential of the genetically modified MSCs and their derived osteoblasts, (1) gene expression profiling, (2) anchorage‐independent growth (AIG) assay in vitro, and (3) tumor formation assay in vivo on the *TP53^+/+^* and two *TP53^−/−^* hESC‐derived MSCs and their derived osteoblasts in comparison with the three OS cell lines (Figure S5G). Principal component analysis (PCA) of RNA‐seq data revealed the proximal clustering of the MSCs (blue) and MSC‐derived osteoblasts (purple) as well as the three OS cell lines (orange) around the 0 level of PC2, indicating high intra‐ and inter‐group similarities. In contrast, hESCs (red) and NCCs (green) formed distinct clusters from the cell types above, indicating their divergent molecular characteristics (Figure 5D).

In vitro AIG assay suggests a progression of malignant potential across the cell models (Figure 5E). Colonies exceeding 50 µm in diameter were quantified as AIG‐positive events in this assay [26]. Statistical analysis of colony diameter and frequency exhibited minimal AIG with the *TP53^+/+^* MSCs (0/13, 0%) and significantly enhanced AIG (8/13, 61.5%) with the *TP53^−/−^*/*RB^−/−^*/*TERT^OE^*/*c‐MYC^OE^* MSCs. As positive controls, the U2OS OS cells showed further increased AIG (12/15, 80%) whereas the Saos2 OS cells displayed the highest AIG (14/14, 100%) (Figure 5F), indicating complete penetrance. Finally, the in vivo tumor formation assay was conducted on osteocytes derived from the *TP53^−/−^*/*RB^−/−^*/*TERT^OE^*/*c‐MYC^OE^* MSCs. Subcutaneous injection of the MSC‐derived osteocytes at 1×10^7^ cells per injection produced measurable tumors at day 20 post‐injection (Figure 5G). As positive controls, the Saos2 and MG63 OS cells achieved 100% tumor incidence following injection of 2×10^6^ cells per injection (Figure 5G,H). Histopathological analysis showed the MSC‐derived tumors somehow recapitulated the histological architecture of MG63 OS per H&E and Picrosirius Red staining (Figure 5I). Expression of OS markers ALP and COL1A1 as detected via immunofluorescence (Figure 5J) indicates that the tumor‐like mass formed by the *TP53^−/−^*/*RB1^−/−^*/*TERT^OE^*/*c‐MYC^OE^* MSC‐derived osteocytes exhibited features of OS. These results suggest that *TP53^−/−^* MSCs can be used to study early events associated with OS development and that additional genetic alterations are required for acquisition of the tumorigenic potential.

### 2.6 *TP53^−/−^* MSCs Resist NK Cell Immunity through IFN‐β‐Mediated HLA‐Ia Up‐Regulation

**FIGURE 6:**
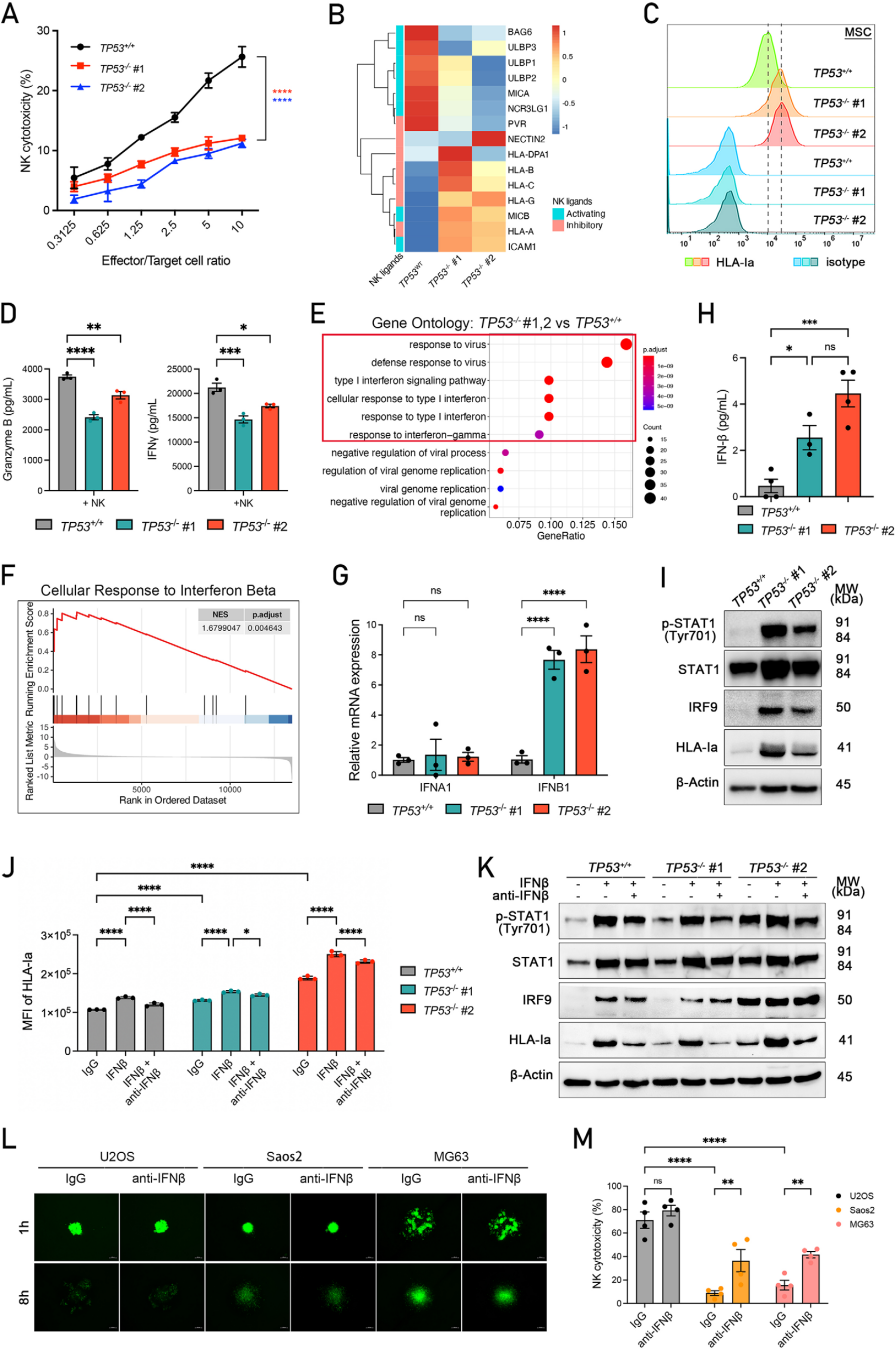
HLA‐Ia upregulation induced by autocrine IFN‐β signaling enhances NK resistance of *TP53^−/−^* MSCs. (A) Human primary NK cell‐mediated cytotoxicity against *TP53^−/−^* and *TP53^+/+^* MSCs. Data are shown as mean ± SEM, *n* = 3, ****p < 0.0001 per ordinary two‐way ANOVA followed by Tukey's multiple comparisons test. (B) Heatmap for expression of NK regulatory ligands in MSCs. (C) Flow cytometry analysis of cell‐surface expression of HLA‐Ia on MSCs. Representative of three independent experiments (*n* = 3). (D) ELISA for granzyme B and IFNγ secreted by primary NK cells. *n* = 3, mean ± SEM, *p < 0.05, ***p < 0.001, ****p < 0.0001 per ordinary one‐way ANOVA followed by Dunnett's multiple comparisons test. (E) GO enrichment of upregulated genes in *TP53^−/−^* MSCs compared to *TP53^+/+^* MSCs. (F) GSEA enrichment of “Cellular Response to Interferon Beta” in *TP53^−/−^* MSCs. (G) qPCR analysis for mRNA level of *IFNA1* and *IFNB1* in MSCs. Data are shown as mean ± SEM, *n* = 3; ns, non‐significance. ****p < 0.0001 ordinary two‐way ANOVA with Šidák's multiple comparisons test. (H) ELISA for IFN‐β secreted by MSCs. *n* = 3, mean ± SEM, *p < 0.05, **p < 0.01 per ordinary two‐way ANOVA followed by Tukey's multiple comparisons test. (I) Immunoblotting analysis for p‐STAT1, total STAT1, IRF9 and HLA‐Ia expression in MSCs. β‐actin was tested as an internal control. (J) Flow cytometry analysis of cell surface HLA‐Ia under IFN‐β or neutralizing IFN‐β antibody treatment. Data are shown as mean ± SEM, *n* = 3. *p < 0.05, ****p < 0.0001 ordinary two‐way ANOVA with Tukey's multiple comparisons test. (K) Immunoblotting analysis for p‐STAT1, total STAT1, IRF9, and HLA‐Ia expression in MSCs under IFN‐β or neutralizing IFN‐β antibody treatment. β‐actin was tested as an internal control. (L, M) Representative live‐cell fluorescence images (L) and a bar chart (M) for NK cell‐mediated cytotoxicity of GFP‐labeled OS cells under IFN‐β or neutralizing IFN‐β antibody treatment. Data are shown as mean ± SEM, *n* = 4, *p < 0.05, **p < 0.01, ****p < 0.0001 per ordinary two‐way ANOVA with Tukey's multiple comparisons test.

Next, we investigate whether *TP53^−/−^* MSCs which, like *TP53^−/−^* OS cells, also exhibit resistance to NK cell‐mediated cytotoxicity. Indeed, *TP53^−/−^* MSCs showed greater resistance to NK cell‐mediated cytotoxicity than *TP53^+/+^* MSCs (Figure 6A). RNA‐seq and qPCR analyses demonstrated increased expression of NK inhibitory ligands and decreased expression of NK activating ligands in *TP53^−/−^* MSCs compared to *TP53^+/+^* MSCs (Figure 6B; Figure S7A,B). Flow cytometry further confirmed higher levels of HLA‐Ia on the cell surface of *TP53^−/−^* MSCs than *TP53^+/+^* MSCs (Figure 6C). Consistently, NK cells secreted lower amounts of granzyme B and IFNγ when co‐cultured with *TP53^−/−^* MSCs than *TP53^+/+^* MSCs (Figure 6D).

As a potent inducer of HLA‐Ia and other NK inhibitory ligands, the type I IFN pathway was highly enriched in *TP53^−/−^* MSCs according to GO analysis of the upregulated genes (Figure 6E) and the enrichment was evidenced through gene set enrichment analysis (GSEA) of the cellular response to IFN‐β (Figure 6F). qPCR analysis showed that *IFNB1*, but not *IFNA*, was significantly increased in *TP53^−/−^* MSCs compared to *TP53^+/+^* MSCs (Figure 6G). Consistently, ELISA analysis exhibited higher IFN‐β secretion by *TP53^−/−^* MSCs than *TP53^+/+^* MSCs, either in the absence (Figure 6H) or presence (Figure S7C) of NK cells. Co‐culture of *TP53^−/−^* MSCs with NK cells was used to mimic the early interactions between transforming MSCs and NK cells during OS initiation.

To examine whether IFN‐β regulates HLA‐Ia expression in MSCs, exogenous IFN‐β was applied at concentrations comparable to those detected above. IFN‐β treatment dose‐dependently increased cell‐surface expression of HLA‐Ia in MSCs (Figure S7D). Western blotting further shows higher levels of phosphorylated STAT1 (p‐STAT1), IRF9, and HLA‐Ia in the *TP53^−/−^* MSCs than *TP53^+/+^* MSCs (Figure 6I). IFNβ blockade with an anti‐IFNβ antibody attenuated STAT1 activation and reversed the IFNβ‐induced increase of cell‐surface HLA‐Ia expression (Figure 6J,K; Figure S7F), indicating the specificity of IFNβ effect. Since TAP1/TAP2 are key components of the antigen‐processing machinery required for peptide transport and stable MHC‐I surface presentation, their expression was also examined. Although TAP2 was up‐regulated in *TP53^−/−^* MSCs (Figure S7E), IFNβ treatment which activated STAT1 signaling (Figure 6K) did not alter TAP1 or TAP2 expression in *TP53^+/+^* MSCs (Figure S7F). The activation of STAT1 and NF‐κB signaling was also observed in MSC‐derived osteoblasts (Figure S7G,H). Together, these findings suggest that IFN‐β contributes to cell‐surface presentation of HLA‐Ia and NK cell resistance in *TP53^−/−^* MSCs and osteoblasts, probably independent of transcriptional induction of *TAP1*/*TAP2*.

### 2.7 Cytosolic dsDNA‐Related NF‐κB Signaling Promotes IFN‐β Expression and HLA‐Ia Mediated NK Cell Resistance in *TP53^−/−^* MSCs

**FIGURE 7:**
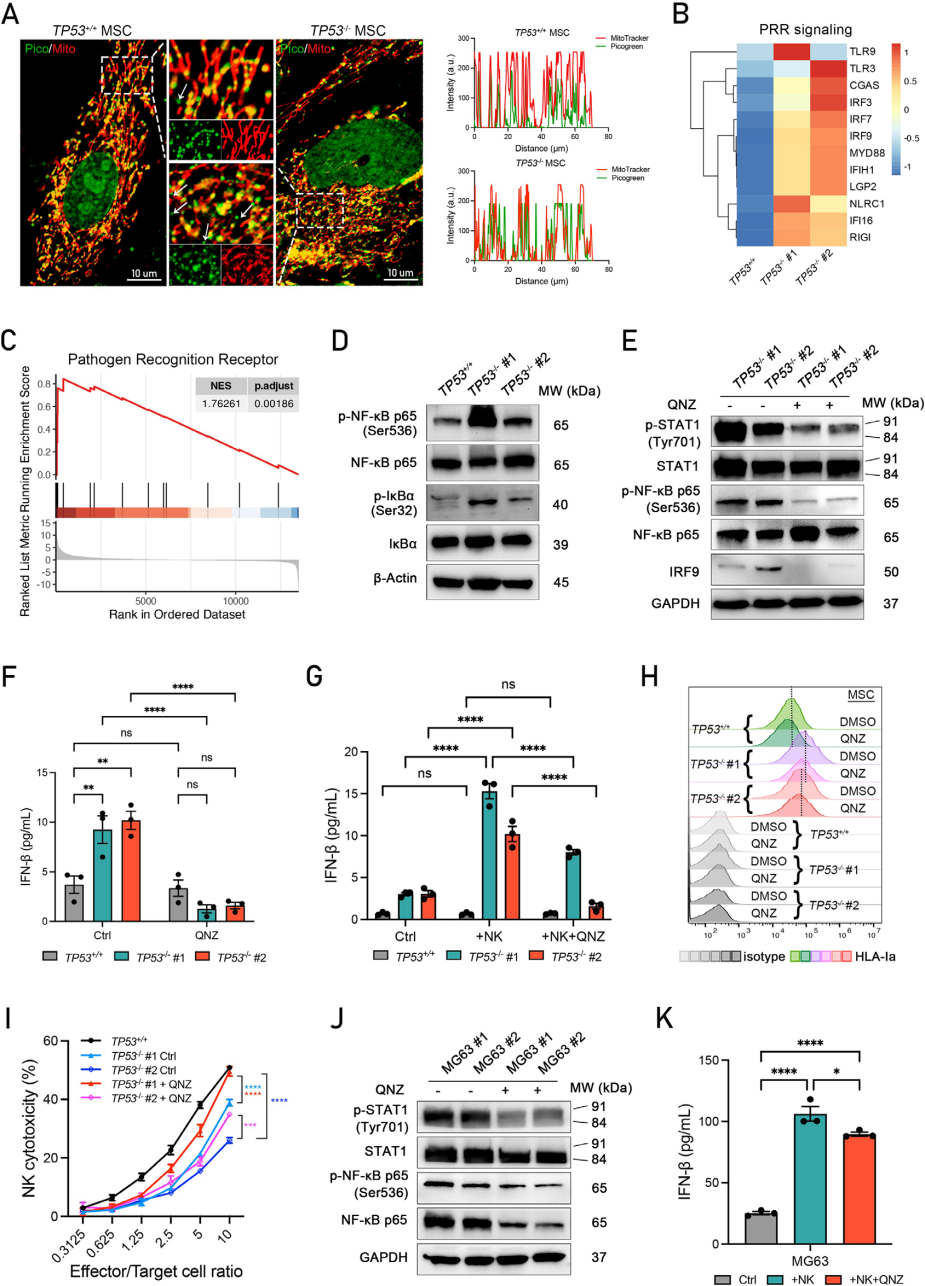
IFN‐β autocrine in *TP53^−/−^* cells is enhanced by cytosolic dsDNA‐activated NF‐κB signaling. (A) Tracking of cytosolic dsDNA in MSCs. dsDNA was stained by Picogreen (green) and mitochondrial DNA by MitoTracker (red), whereas dsDNA (green) that is free in the cytosol is recognized as cytosolic dsDNA (left panel). The staining is illustrated in colocalization plots (right panel). Scale bar: 10 µm. (B) Heatmap for expression of genes related to PRR signaling in *TP53^−/−^* versus *TP53^+/+^* MSCs. (C) GSEA enrichment of PRR signaling genes in *TP53^−/−^* versus *TP53^+/+^* MSCs. (D) Immunoblotting for designated proteins in *TP53^−/−^* versus *TP53^+/+^* MSC. β‐actin was tested as an internal control. (E–I) Immunoblotting for designated proteins (E), ELISA for IFN‐β secretion in the absence (F) and presence (G) of primary NK cells, flow cytometry analysis of cell‐surface HLA‐Ia (H), and NK cell‐mediated cytotoxicity (I) for *TP53^+/+^* versus *TP53^−/−^* MSCs with or without treatment of QNZ at 10 nm for 24 h. Data are shown as mean ± SEM, *n* = 3, **p < 0.01, ***p < 0.001, ****p < 0.0001 per RM two‐way ANOVA followed by Tukey's multiple comparisons test. ns: non‐significant. (J, K) Immunoblotting for designated proteins in MG63 cells (J) and ELISA for IFN‐β secretion by MG63 cells (K) in the presence of primary NK cells and with or without QNZ treatment. For K, data are shown as mean ± SEM, *n* = 3, *p < 0.05, ****p < 0.001 per ordinary one‐way ANOVA with Tukey's multiple comparisons test.

The type I IFN signaling can be activated upon recognition of damage‐associated molecular patterns (DAMPs) by pattern recognition receptors (PRRs). DAMPs include various forms of cytosolic nucleic acids, such as double‐stranded (ds) or single‐stranded (ss) DNA and RNA [56]. Because p53 loss or dysfunction can promote genomic instability and accumulation of cytosolic nucleic acids, we investigated whether this mechanism underlies the induction of IFN signaling in *TP53^−/−^* MSCs. The presence of cytosolic DNA was examined in MSCs with cytosolic versus total dsDNA stained with Picogreen (green) and mitochondria with MitoTracker (red). Apart from the nucleus and mitochondria, very little cytosolic dsDNA was found in *TP53^+/+^* MSCs. In contrast, increased cytoplasmic dsDNA was detected in *TP53^−/−^* MSCs (Figure 7A, left), as further illustrated by colocalization analysis of the stained DNA (Figure 7A, right). Consistently, genes encoding PRRs, including *TLR9* and *TLR3*, the dsDNA sensor *CGAS*, and dsRNA/ssRNA sensors *RIGI* and *IFI16* were upregulated in *TP53^−/−^* MSCs compared to *TP53^+/+^* MSCs. Similarly, genes involved in the type I IFN signaling, including *STAT1*, and *STAT2*, *IRF3*, *IRF7*, and *IRF9*, were also upregulated (Figure 7B and Figure S8A). GSEA analysis further exhibited positive enrichment of the PRR‐related genes in *TP53^−/−^* MSCs compared with *TP53^+/+^* MSCs (Figure 7C).

Cytosolic dsDNA sensing can induce IFN expression through TBK1‐dependent pathways or IKK‐NF‐κB pathways [57]. NF‐κB can also regulate *IFNB* expression independently of IRF3 under certain conditions [58]. In *TP53^−/−^* MSCs, increased levels of phosphorylated IκB (p‐IκB) and phosphorylated NF‐κB (p‐NF‐κB) were observed (Figure 7D), along with elevated cGAS expression, whereas the expression and phosphorylation levels of STING, TBK1, and IRF3 were not markedly increased compared with *TP53^+/+^* MSCs (Figure S8B). These results suggest that p53 loss is associated with activation of a cytosolic dsDNA‐driven NF‐κB signaling program in *TP53^−/−^* MSCs. To assess the functional role of NF‐κB in IFN‐β production, HLA‐Ia upregulation, and NK resistance, a selective NF‐κB inhibitor quinazoline (QNZ) [59] was employed in the following assays. QNZ treatment reduced levels of p‐NF‐κB, phosphorylated STAT1 (p‐STAT1), and IRF9 (Figure 7E), accompanied by decreased IFN‐β secretion in both *TP53^+/+^* and *TP53^−/−^* MSCs either in the absence (Figure 7F) or presence (Figure 7G) of NK cells. QNZ also reduced cell‐surface HLA‐Ia expression (Figure 7H) and increased sensitivity to NK cell‐mediated cytotoxicity (Figure 7I) in *TP53^+/+^* or *TP53^−/−^* MSCs. QNZ also inhibited *IFNB1* expression in both MSCs at the transcriptional level (Figure S8C).

To determine whether similar signaling features were present in OS cells, the NF‐κB and STAT1 signaling in the OS cell lines were examined. Among them, MG63, an immature osteoblast‐like OS cell line [29], expressed higher p‐STAT1 level (Figure S8D) and secreted more IFN‐β than U2OS and Saos2, particularly in the presence of NK cells (Figure S8E). QNZ treatment reduced p‐NF‐κB and p‐STAT1 levels (Figure 7J) and NK cell‐induced IFN‐β secretion (Figure 7K) in MG63 cells. Together, these findings suggest that p53 loss is associated with activation of cytosolic dsDNA‐NF‐κB‐IFN‐β‐HLA signaling in MSCs and OS cells, which may contribute to HLA‐Ia‐mediated NK cell resistance.

### 2.8 Inverse Relationship between p53 Function and HLA‐Ia Expression Is Validated in Human Osteosarcoma Samples

**FIGURE 8:**
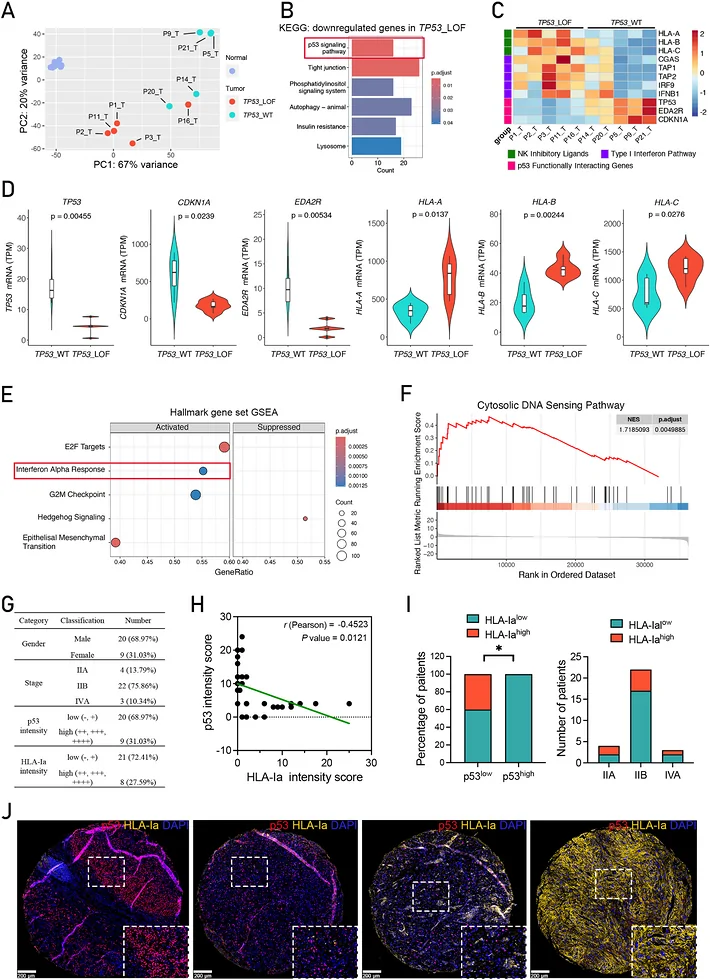
Inverse relationship between p53 and HLA‐Ia is validated in human osteosarcoma samples. (A) PCA for *TP53*_LOF versus *TP53*_WT OS samples from patients and paired normal controls. (B) KEGG enrichment of downregulated genes in the *TP53*_LOF OS samples. (C) Heatmap for p53 function‐associated genes, type‐I IFN associated genes, and NK inhibitory ligand genes in the OS samples. (D) Expression levels of p53 function‐related genes and HLA‐Ia genes in *TP53*_WT and *TP53*_LOF OS specimens from the GSE126209 dataset. Data are shown as TPM values. Statistical significance was determined using an unpaired Student's *t*‐test. (E) GSEA enrichment of hallmark gene sets in the OS samples. (F) GSEA enrichment of cytosolic DNA sensing pathway in the OS samples. (G) Clinical information of the human OS tissue array specimens. (H) Correlation analysis between p53 and HLA‐Ia staining intensity scores in the human OS tissue array specimens. (I) Left, distribution of HLA‐Ia^low^ and HLA‐Ia^high^ samples in the p53^low^ and p53^high^ groups. Statistical significance was determined using Fisher's exact test. Right, distribution of HLA‐Ia^low^ and HLA‐Ia^high^ samples across OS stages. (J) Representative multiplex immunofluorescence images showing p53 and HLA‐Ia staining. Magnifications show at lower right corner. Scale bars, 200 µm.

To assess clinical relevance of the findings from the in vitro and animal models, we analyzed the OS dataset GSE126209 which contains both tumor tissues and paired normal tissues [60]. Tumor samples were classified into *TP53*_WT and *TP53*_LOF groups by comparing the transcript reads at the *TP53* locus in the binary alignment map (BAM) data of the tumor tissues and their paired normal tissues (Figure S9). For instance, patient #9 showing abundant *TP53* reads without detectable mutation in both tumor and paired normal tissue, was classified as *TP53*_WT (Figure S9A). In contrast, patient #3 showing loss of *TP53* reads in tumor tissue but retained *TP53* reads in paired normal tissue, was classified as *TP53*_LOF (Figure S9B). Principal component analysis (PCA) revealed clear separation between the *TP53*_WT and *TP53*_LOF samples (Figure 8A). Consistent with this classification, KEGG pathway analysis showed that genes downregulated in the *TP53*_LOF group were significantly enriched for the p53 signaling pathway, supporting the validity of *TP53*_WT/*TP53*_LOF stratification (Figure 8B). Heatmap analysis further revealed reduced p53 functional interactors, accompanied by increased expression of type I IFN pathway‐associated genes and HLA‐Ia genes in the *TP53*_LOF group (Figure 8C). In contrast, *TP53*_WT samples exhibited higher expression of p53 function‐related genes, including *TP53*, *CDKN1A*, and *EDA2R*, but lower expression of HLA‐Ia genes (Figure 8D). Furthermore, GSEA revealed activation of the type I IFN response in the *TP53*_LOF group (Figure 8E), accompanied by enrichment of the cytosolic DNA sensing pathway (Figure 8F).

To further validate this relationship in clinical specimens, p53 and HLA‐Ia expression were assessed in a human OS tissue array. Patient characteristics, including gender and OS stage, are summarized in Figure 8G. Correlation analysis based on p53 and HLA‐Ia staining intensity scores revealed a negative association between p53 and HLA‐Ia expression within this cohort (Figure 8H). Fisher's exact test further demonstrated that HLA‐Ia expression was infrequently detected in the p53^high^ group, whereas p53 expression was rarely observed in the HLA‐Ia^high^ group (Figure 8I, left). However, likely due to the limited sample size and the absence of early‐stage OS specimens, no significant differences in HLA‐Ia distribution were observed among tumors at different stages (IIA, IIB, and IVA) (Figure 8I, right). Representative images of p53 and HLA‐Ia staining are shown in Figure 8J. Collectively, these analyses of clinical OS samples support an inverse relationship between p53 expression/function and HLA‐Ia expression in human OS.

## 3 Discussion

In this study we have provided evidence that p53 loss is associated with increased NK cell resistance in OS cells and establish an MSC‐based model to investigate early events associated with OS development, which involves a cytosolic dsDNA‐triggered NF‐κB‐IFN‐β‐HLA‐Ia signaling axis. The tumor suppressor p53 in both WT and mutated forms can modulate cancer cell responses to immune cells including NK cells in distinctive ways [61]. These effects vary depending on the status of p53 functions, the type and stage of tumors, and the cell types that harbor the p53 mutations [18]. While the tumorigenic role of p53 loss in OS development has been well‐documented [1], how it regulates NK cell responses of OS cells remains elusive or even controversial.

Wang, et al. reported that *TP53^+/+^* human OS U2OS cells express slightly higher cell‐surface HLA‐Ia than *TP53^−/−^* Saos2 cells, which involves the direct binding of p53 to *ERAP1* [19], a gene crucial for processing and presenting peptides on HLA‐Ia. The authors identified two potential p53 responsive elements in an intron of *ERAP1* using 5‐fluorouracil‐treated peripheral blood mononuclear cells and then demonstrated p53 binding to the region linked to a luciferase reporter [19]. Furthermore, the authors found that a p53 activator nutlin‐3a, which inhibits MDM2 to prevent p53 degradation, can increases HLA‐Ia expression in *TP53^+/+^* cells [19]. They concluded that WT p53 upregulates HLA‐Ia by binding and inducing *ERAP2* expression. Interestingly, by using the same OS cell lines plus another *TP53^−/−^* OS cell line MG63, we obtained opposite results that *TP53^−/−^* OS cell lines expressed cell‐surface HLA‐Ia much higher than the *TP53^+/+^* U2OS cell line. Similar results were observed with *TP53^−/−^* MSCs, compared to their isogenic WT control. These differences may be attributed to the following factors. (1) Both assays Wang and coworkers used for p53‐*ERAP2* binding are not directly relevant to the pathological conditions in tumor cells. Such binding was not found according to our analysis of a ChIP‐seq database on etoposide‐treated U2OS cells (data not shown) [62]. Besides, the ChIP‐seq assay did not detect p53 binding to the *ERAP1* gene in *TP53^+/+^* MSCs (data not shown); (2) Since MDM2 degrades not only p53 but other proteins including retinoblastoma protein, E2F1, and FOXO3a [63], inhibiting MDM2 with nutlin‐3a may elevate not only p53 but also the other proteins. Thus, the slight increase of cell‐surface HLA‐Ia expression may result from a combinatory effect of all these upregulated target proteins.

It is worth noting that p53 loss has also been reported to be associated with aberrant IFN‐I signaling and premalignant transformation during chronic myeloid leukemia development [64]. However, the role of MHC and NK cell resistance in this pathological process was not revealed in this report. Although we confirmed that IFN‐I signaling is responsible for the NK resistance during OS initiation, additional signals, e.g., those from the epithelial‐mesenchymal transition (EMT) cannot be excluded as they can alter or fine‐tune the MHC‐I level in other cancer types. For example, TGF‐β‐induced EMT in non‐metastatic lung carcinoma cells led to upregulation of MHC‐I and TAP1, whereas metastatic cells showed downregulation [65]. Furthermore, in breast cancer cell MCF7 cells, β‐estradiol decreased MHC‐I expression, while fulvestrant (an ER antagonist) and IFN‐γ increasing it [66]. Thus, different mechanisms might involve in MHC‐I regulation, depending on dominant signals or their concerted effect in the tumor microenvironment.

As for mutations of *TP53*, they are also frequently found in OSs and are strongly associated with the development of the mesenchymal tumor [67]. Mice with *Trp53* (equivalent to human *TP53*) loss are extremely susceptible to radiation‐induced OSs [68]. *Trp53* knockout in mouse mesenchymal stem cells (MSCs) causes the development of undifferentiated soft tissue sarcoma [21]. Furthermore, OSs were observed in patients with Li‐Fraumeni syndrome, which is characterized by germline *TP53* inactivation [69]. To confirm MSCs as such a source so that they can be used to model early OS development and dissect the underlying mechanism, we analyzed scRNA‐seq data of OSs from 17 patients across various pathological conditions. We found that OS‐contained MSCs among all the samples consistently expressed an immunophenotype of CD44^+^CD73^+^CD90^+^CD105^+^, identical to that of MSCs in vitro, but distinct from that of MSCs (ALDH1A1^+^KLF4^+^LEPR^+^) in breast cancer [70] and MSCs (CA1^+^ATPIF1^+^AHSP^+^S1009A^+^) in normal spongy bone tissue [71]. Furthermore, the OS MSCs carried high CNV burden like osteoblasts, and MSCs with lower p53 function displayed fewer signal communications with immune cells including NK cells than MSCs with higher p53 function. These data support the appropriateness of *TP53^−/−^* MSCs for modeling early cellular states associated with OS development.

Following dissection of the signaling pathways in *TP53^−/−^* MSCs in comparison with *TP53^+/+^* MSCs, we found that IFN‐β upregulation occurred independently of the canonical cGAS‐STING signaling in *TP53^−/−^* MSCs. The apparent absence of STING activation despite cGAS upregulation likely stems from the intrinsic incapacity of MSCs to synthesize cGAMP required for STING activation, as reported by others [72] and ourselves [73]. Further mechanistic studies revealed that NF‐κB directly induced IFN‐β production, instead of through the STING‐TBK1‐IRF3 signaling. It has been known that p53 can regulate innate immunity through many other mechanisms, for example, by directly inducing the expression of *TLR3* [74], *TLR5* [75], and IRFs [76] in HCT116 colon cancer and MCF7 breast cancer cells. However, p53 binding to these genes was not detected in *TP53^+/+^* MSCs in the presence of primary NK cells (data not shown). These differences highlight the diversity of p53 functions in different cell types.

Previous studies have reported variable HLA expression in OS [77, 78], and the present study also revealed variable HLA‐Ia expression across different stages of OS. These variations may have important implications for immunotherapy. HLA loss may facilitate escape from CD8^+^ T cell recognition, whereas retained HLA expression in metastatic high‐grade OS has been associated with increased T cell infiltration and PD‐L1 expression, supporting the relevance of HLA status to T cell‐based immunotherapy [79]. Conversely, increased HLA‐Ia, as observed in *TP53*‐deficient OS cells in this study, may suppress NK cell activity through inhibitory receptor signaling. Therefore, integrated assessment of *TP53* status, HLA‐Ia expression, and NK/CD8^+^ T cell immune contexture may help guide patient stratification and rational combination immunotherapy in OS. For tumors with low HLA‐Ia expression, strategies that enhance CD8‐independent cytotoxicity, such as NK or CAR‐NK cell therapy, may be considered. For tumors with deficient *TP53* signaling/functions and high levels of HLA‐Ia expression, NK cell‐based therapies may require combination with approaches that relieve HLA‐mediated NK inhibition, including blockade of IFN‐β signaling, interruption of HLA and NK cell interactions, or HLA‐neutralizing strategies [80].

In summary, our study identifies p53 loss as an inducer of NK cell immune resistance during OS development. Using OS cell lines, syngeneic mouse models, human clinical samples, and hESC‐derived MSC models, we show that p53 deficiency promotes activation of the cytosolic dsDNA‐NF‐κB‐IFN‐β pathway, leading to increased HLA‐Ia expression and resistance to NK cell‐mediated cytotoxicity. These findings highlight a previously underappreciated role of p53 in regulating innate immune surveillance during early OS development and suggest that targeting the IFN‐β‐HLA‐Ia axis may enhance NK cell‐based therapeutic strategies in TP53‐deficient OS. Further studies are warranted to determine whether targeting the cytosolic dsDNA‐NF‐κB‐IFN‐β‐HLA‐Ia signaling, combined with conventional therapies, could improve the therapeutic responses in OS patients with p53 deficiency and HLA upregulation.

## 4 Material and Methods

### 4.1 Cell Culture

Human osteosarcoma cell lines U2OS, Saos2, and MG63 were from the Cell Bank of the Chinese Academy of Sciences. The mouse osteosarcoma K7M2 cell line was from Procell Life Science & Technology. U2OS cells were cultured in McCoy's 5a (modified) medium (GIBCO) supplemented with 10% fetal bovine serum (FBS) (GIBCO), 1x MEM Non‐Essential Amino Acids solution (NEAA; GIBCO), and 1x GlutaMAX Supplement (GIBCO). For Saos2 cells, the basal medium was identical to that of U2OS cells but contained 15% FBS. MG63 and K7M2 cells were maintained in the Dulbecco's modified Eagle medium (DMEM) (GIBCO) with 10% FBS, 1x NEAA, and GlutaMAX. The H9 hESCs [48] were maintained in our laboratory and cultured in mTeSR1 (STEMCELL Technologies) on the Matrigel‐coated plates at 37°C and in 5% CO_2_. Cells were passaged every 4–5 d, with daily medium replacement. Human leukemia cell line K562 was a gift courtesy of Qi Zhao laboratory, University of Macau. K562 cells were maintained in RPMI‐1640 (GIBCO) supplemented with 10% FBS, 1x NEAA, and GlutaMAX. All cells were tested free of mycoplasma and bacteria contamination.

### 4.2 Mesenchymal Stem Cell Differentiation and Tri‐Lineage Differentiation

Differentiation of hESCs into NCCs was induced using a NCC medium containing essential 6 (E6) medium supplemented with 10 ng mL^−1^ basic fibroblast growth factor (bFGF), 1 µm CHIR99021, 10 µm SB431542, 1 µm dorsomorphin, and 22.5 µg mL^−1^ sodium heparin. After 15 d of induction, cells were validated for NCC‐specific markers HNK‐1 and NGFR‐p75 via immunofluorescence staining and flow cytometry. Subsequently, NCCs further differentiated into MSCs in α‐MEM basal medium containing 20% FBS, 1x NEAA, and GlutaMAX. The MSC identity was confirmed using the BD Stemflow Human MSC Analysis Kit, which detected the expression of positive markers CD44, CD73, CD90, and CD105 and absence of negative markers CD34, CD11b, CD19, CD45, and HLA‐DR. To assess multipotency, tri‐lineage differentiation toward osteogenic, chondrogenic, and adipogenic lineages was performed using the MesenCult Osteogenic, Chondrogenic, and Adipogenic Differentiation Kits (STEMCELL Technology), respectively. Differentiation outcomes were evaluated by histochemical staining of Alizarin Red S for mineralization (osteogenesis), Alcian Blue for glycosaminoglycan deposition (chondrogenesis), and Oil Red O for lipid droplet visualization (adipogenesis).

### 4.3 *TP53*, *RB1*, *B2M*, and *Trp53* KO via CRISPR‐Cas9‐Mediated Genome Editing, and p53 Overexpression

Single‐guide RNAs (sgRNAs) targeting exon 4 of *TP53*, exon 1 of *RB1*, exon 1 of *B2M*, and exon 5 of *Trp53* were designed using the Broad Institute Genetic Perturbation Platform (GPP) sgRNA design tool (https://portals.broadinstitute.org/gpp/public/analysis‐tools/sgrna‐design). The *TP53*‐targeting sgRNA sequences were as follows: top strand 5’‐GCAGTCACAGCACATGACGG‐3’, bottom strand 5’‐CCGTCATGTGCTGTGACTGC‐3’; The *RB1*‐targeting sgRNA sequences were: top strand 5’‐CGGAGGACCTGCCTCTCGTC‐3’, bottom strand 5’‐GACGAGAGGCAGGTCCTCCG‐3’; The B2M‐targeting sgRNA sequences were: top strand 5’‐CGCGAGCACAGCTAAGGCCA‐3’, bottom strand ‐5’ TGGCCTTAGCTGTGCTCGCG ‐3’; The *Trp53*‐targeting sgRNA sequences were: top strand 5’‐ACCCTGTCACCGAGACCCC‐3’, bottom strand 5’‐GGGGTCTCGGTGACAGGGT‐3’; The non‐target gRNA sequences (ntgRNA) were: top strand 5′‐GCGAGGTATTCGGCTCCGCG‐3′, bottom strand 5′‐CGCGGAGCCGAATACCTCGC‐3′. *TP53*, *RB1*, and *Trp53* targeting sgRNA expression plasmids were constructed by ligating these sequences into a PX458 backbone (Addgene #48138) vector containing an EF1α promoter, an E6‐sgRNA scaffold, and an EGFP reporter, following published protocols [81]. ntgRNA and *B2M* targeting sgRNA were ligated into LentiCRISPR v2 (Addgene #52961) backbone vector. hESCs were transfected with the sgRNA plasmids using Lipofectamine Stem Transfection Reagent (Thermo Scientific), and GFP‐positive cells were isolated 48 h post‐transfection via fluorescence‐activated cell sorting (FACS). Single‐cell clones were expanded under continuous monitoring with the IncuCyte ZOOM live‐cell imaging system. Successful KO of *TP53* and *RB1* was validated by Sanger sequencing of target loci and immunoblotting for protein depletion. P53, TERT and c‐MYC overexpression were conducted by lentivirus expression vector PLX313‐TP53‐WT (Addgene #118014), pCDH‐3xFLAG‐TERT (Addgene #51631), c‐MYC‐EGFP (GeneCopeia #EX‐Z2854‐Lv165), and the control vector was PLX313‐Firefly luciferase (Addgene #11801), EGFP (GeneCopeia #EX‐NEG‐Lv165). Transduced cells were selected with associated antibiotics.

### 4.4 Preparation of Metaphase Chromosomes for Karyotyping

The preparation of metaphase chromosomes for hESCs followed publicly available protocols [82]. hESCs were plated in Matrigel‐coated plates and allowed to proliferate until reaching ≈70% confluency. Metaphase arrest was induced by treating cells with 0.1 µg mL^−1^ KaryoMAX Colcemid solution (Gibco) in mTeSR1 medium at 37°C for 2 h. Following medium removal, cells were washed twice with Dulbecco's phosphate‐buffered saline (DPBS). Detached cells were collected into the complete medium, and centrifuged at 1000 rpm for 5 min. The supernatant was carefully aspirated, retaining 0.5 mL residual medium to prevent cell clumping. 8 mL of pre‐warmed 0.075 m KCl was added into cells and incubated at 37°C for 20 min. The reaction was stopped with 1 mL of freshly prepared fixative, methyl alcohol/glacial acetic acid at the 3:1 (volume to volume) ratio, and the metaphase chromosomes were stocked in the fixative at −20°C prior to slide preparation for chromosome analysis.

### 4.5 Primary NK, CD8^+^ T Cell Culture and NK, CD8^+^ T Cell‐Mediated Cytotoxicity Assays

Primary NK cells were isolated from human peripheral blood mononuclear (PBMCs) using established protocol [73]. Briefly, PBMCs were co‐cultured with γ‐irradiated (100 Gy) K562 cells genetically engineered to express membrane‐bound interleukin‐21 (mIL‐21) as artificial antigen‐presenting cells (aAPCs) [83]. The cells were maintained in RPMI‐1640 with 10% FBS, 200 U mL^−1^ IL‐2 (PeproTech) and 5 ng mL^−1^ IL‐15 (PeproTech) at 37°C in 5% CO_2_. Primary T cells were isolated from PBMCs using CD3 MicroBeads (Miltenyi Biotec, 130‐097‐043) and CD8^+^ T cells were activated and expansion using Dynabeads Human T‐Activator CD3/CD28 (Gibco, 11161D) following the manufacturer's protocol. CD8^+^ T cells were maintained in RPMI‐1640 with 10% FBS, 30 U ml^−1^ IL‐2 (PeproTech) at 37°C in 5% CO_2_.

For in vitro NK cytotoxicity assay, calcein‐AM was used to label 1 × 10^4^ MSCs or OS cells as target cells in the corresponding target cell medium. The cell suspension was aliquoted into 96‐well microplates (100 µL per well). Primary NK cells as effector cells were then added into the wells of the microplates at effector‐target (E/T) ratios of 10:1, 5:1, 2.5:1, 1.25:1, 0.625:1, and 0.3125:1 in triplicate. Technical controls included (1) target cells alone with 1% Triton X‐100 treatment as the maximum release group and (2) target cells alone without any treatment as the spontaneous release group. After 4 h incubation, the 535 nm absorbance was recorded by a microplate reader. For visible tracking of NK or CD8^+^ T cell cytotoxicity, target cells with GFP label (3000 cells per well in 100 µL media) were plated into clear round bottom ultra‐low attachment 96‐well microplate. Primary NK cells (10 000 cells per well in 100 µL NK cell media) or CD8^+^ T cells (10000 cells per well in 100 µL T cell media) were added. Photos were taken every 4 or 12 h. Remaining signals were calculated based on the percentage of the GFP signal at the photo time point compared to the initial time point.

In vivo NK cytotoxicity assay employed two models. (1) Intraperitoneal model: 2 × 10^5^ Luc‐expressing target cells were co‐injected i.p. with 4 × 10^6^ primary NK cells into the 4–6 weeks old NOD/SCID mice (*n* = 4 per group). Bioluminescence was quantified on day 0 and 4 post‐injection following i.p. administration of D‐Luciferin at 15 mg mL^−1^ for 150 mg kg^−1^. (2) Subcutaneous model: 5 × 10^6^ target cells were mixed with primary NK cells at a 1:5 E/T ratio. The mixture was implanted subcutaneously (s.c.) into 4–6 weeks old NOD/SCID mice. Tumors were excised at day 21 post‐inoculation.

### 4.6 Teratoma Assay

The teratoma assay experiments were approved by the Animal Research Ethics Committee of the University of Macau under the protocol #UMARE‐011‐2019. The procedure of teratoma assay followed a previous study [48]. 1 million hESCs were suspended with 100 µL of mixture of Matrigel and DPBS buffer at a 1:1 ratio and the cell suspension was s.c. injected into the hind limb of 4–6 weeks old NOD/SCID mice. Six to eight weeks after the injection or when the tumor reached 1 cm in diameter, the mice were euthanized in a CO_2_ chamber. Teratomas were collected and fixed by 4°C 4% paraformaldehyde for overnight, followed by tissue processing and hematoxylin and eosin (H‐E) staining.

### 4.7 Anchorage‐Independent Growth (AIG) Assay

Soft‐agar colony formation assay was used to evaluate the AIG of premalignant and malignant cells. It included three layers, i.e., the top, middle, and bottom layers [26]. The bottom layer was formed by 1.5 mL of 0.6% low‐melting agarose (Fisher Scientific) mixed with the target cell complete medium and solidified in a six‐well plate. The middle layer, plated on the bottom layer, was formed by a suspension of 5000 cells in 1.5 mL of 0.3% low‐melting agarose with the target cell complete medium. The top layer was 1 mL target cell complete medium added on the top of the middle layer. Cells in agarose plated were incubated at 37°C in 5% CO_2_ and photos were taken weekly. Only colonies exceeding 50 µm in diameter were quantified as AIG‐positive events.

### 4.8 Xenogeneic, Syngeneic Graft Models, and Orthotopic Osteosarcoma Model

These experiments were approved by the Animal Research Ethics Committee of the University of Macau under the protocol #UMARE‐016‐2020, #UMAR‐032‐2016. MSC‐derived osteocytes (1×10^7^ cells per injection) and OS cells (2×10^6^ cells per injection) were suspended in a mixture of Matrigel: DPBS at 1:1 ratio. The cell suspension was s.c. injected into 4–6 weeks NOD/SCID mice. Samples were collected at days 20 (for MSC‐derived osteocytes group) or 21 (for OS group) post‐injection. Luciferase‐expressing K7M2 cells were suspended in a mixture of Matrigel: DPBS at 1:1 ratio and then inoculated into 6 weeks‐old BALB/c mice at 5×10^6^ cells per subcutaneous injection. The animals and tumorigenesis were monitored every day. Xenografts were excised at days 7 post‐injection. Tissues were fixed at 4°C in 4% paraformaldehyde overnight and processed for histology and immunostaining. Human or murine OS cells (1×10^6^ cells per injection in 30 µL DPBS) were administrated through intratibial injection in the orthotopic OS model.

### 4.9 Cytosolic dsDNA Staining

10^4^ cells were plated into a 35 mm confocal dish one day before staining. Cells were incubated with 3 µL mL^−1^ Picogreen (Maokang Biotechnology) at 0.1 mg mL^−1^ for 30 min. to stain dsDNA. Then, 1 µL mL^−1^ MitoTracker (Beyotime) was added in for another 30 min. to stain the mitochondria DNA. After washing with DPBS, Hoechst 33342 solution at 20 mm was added to stain the cell nuclei. The images of stained dsDNA, mitochondria DNA, and nuclei were obtained through confocal laser scanning microscope (Carl Zeiss LSM 880).

### 4.10 HLA and KIR Typing

HLA typing was performed based on the paired‐end RNA‐seq data using arcasHLA according to a previous study [84]. Briefly, coordinate‐sorted BAM files were first processed with arcasHLA extract to obtain HLA‐informative reads. The extracted paired‐end FASTQ files were then analyzed with arcasHLA genotype against an IMGT/HLA‐derived transcript reference (version 3.24.0), and HLA class I alleles were inferred from the allele pairs best supported by the pseudoaligned reads. KIR genotyping was performed using genomic DNA extracted from NK cells according to a previously described real‐time quantitative PCR‐based method [85]. Briefly, locus‐specific primers targeting individual KIR genes were used for SYBR green‐based qPCR amplification. Gene positivity was determined according to Ct values, and only reactions showing a single specific melting‐curve peak were considered valid. KIR3DL3 was used as an internal reference gene. The resulting KIR presence/absence profiles were used to define the KIR genotype of each sample.

### 4.11 Chromatin Immunoprecipitation Sequencing (ChIP‐Seq)

First, ChIP was conducted following the publicly available protocols [86]. Briefly, cells were plated into 10 cm dishes and cultured in a corresponding medium until 90% density. 62.5 µL of 16% (wt/vol) formaldehyde (Thermo Fisher Scientific) per 1 mL medium was added to the dishes, followed by incubation at 37°C on a slow shaker for 10 min. Formaldehyde was quenched with 2 m ice‐cold glycine at 66.4 µL per 1 mL (for the final concentration of glycine in 125 mm) for 10 min. at room temperature. After removal of the solutions, the cells were washed with ice‐cold DPBS twice, scraped, and collected into Eppendorf tubes for centrifugation. Cell pellet was lysed and sonicated in a low salt IP buffer and then incubated with pre‐cleaned ChIP‐Grade Protein A/G Plus Agarose beads (Thermo Fisher Scientific) at 4°C for 1 h. The supernatant was collected for incubation with anti‐p53 antibody (Santa Cruz, sc‐126) at 4°C overnight with constant rotations. After that, second round of pre‐cleaned ChIP‐Grade Protein A/G Plus Agarose beads was added and incubated at 4°C for 3 h with rotations. Then, beads were collected and incubated with a resuspension buffer at 65°C overnight with shaking at 800 rpm. Supernatant was collected for DNA isolation using UltraPure Phenol:Chloroform:Isoamyl Alcohol (Invitrogen). All the procedures were conducted according to the instructions of manufacturer. The final ChIP DNA were sent for DNA sequencing with the Illumina HiSeq platform.

### 4.12 Bioinformatic Analysis

The raw count matrices of scRNA‐seq data (GSE152048, GSE162454) were acquired from the NCBI Gene Expression Omnibus (GEO) database repository. Data processing was performed in R (v4.3.2) by using the Seurat package (v4.4.0). Cells meeting the following criteria were retained: (1) detection of >200 unique genes (nFeature_RNA) and (2) mitochondrial gene contribution at <10% (percent.mt). First, the LogNormalize method was used for the normalization of raw count data. Next, the FindVariable_Features function was used to calculate the top 2000 highly variable genes. The principal component (PC) calculations were performed according to the top 2000 highly variable genes. The integration and batch correction were conducted using Harmony algorithm. The cell clustering was performed using FindNeighbors and FindClusters functions. Finally, the UMAP plot was used to visualize the cell clustering. CellChat (v2.1.2) was utilized to analyze and visualize the cell–cell communications.

In‐house RNA‐seq was conducted on the Illumina HiSeq Sequencer. For high data quality, raw data were cleaned using the trim_glore software. Qualified filtered data were generated using FastQC (v0.10.1) for naked‐eye inspection. Then reads were aligned to human reference genome hg38 (GRCh38.p12) using the STAR software (v2.7.0f) and gene counts were calculated using the FeatuteCounts software separately. The raw count data were imported to R language for further exploration. The transcript per million (TPM) was calculated based on the sequence depth and gene length, and normalization of TPM was used for PCA and heatmap visualization. For analysis of differentially expressed genes (DEGs), DESeq2 (v1.38.3) and EdgeR (v3.40.2) were used according to different experimental design. The DEGs were defined based on the log 2‐fold change (log2FC), p.adj, and *p* value. For DESeq2 results, the threshold is log2FC > 1 and false discovery rate (FDR) < 0.05; for EdgeR results, the thresholds were log2FC > 1 and *p* value < 0.01. The gene ontology (GO) enrichment analysis was performed using the ClusterProfiler package (v4.6.2) and visualizations were conducted using the enrichplot package (v1.18.3). Heatmap visualizations were generated using the pheatmap (v1.0.12) and ggplot2 (v3.4.0) package. All the raw data and associated metadata generated through next‐generation sequencing in this study are available upon request.

## Author Contributions

**Guihui Qin**: conceptualization, investigation, validation, methodology, software, formal analysis, data curation, project administration, visualization, Writing – original draft, Writing – review and editing. **Cheung Kwan Yeung**: methodology, data curation, investigation, writing – review and editing. **Ye Yi**: methodology, data curation, writing – review and editing, investigation. **Dejin Zheng**: conceptualization, methodology, writing – review and editing, visualization. **Miaoman Ye**: methodology, data curation. **Chi‐Chong Chio**: data curation, writing – review and editing. **Jinjie Wu**: data curation, writing – review and editing. **Siyi Fu**: writing – review and editing. **Chu‐Xia Deng**: resources. **Ren‐He Xu**: conceptualization, supervision, project administration, resources, funding acquisition, writing – review and editing.

## Ethics Statement

This study strictly followed the International Society for Stem Cell Research guidelines for studies on hESCs. The H9 hESC line used here was registered as an ethically approved line at the National Institutes of Health. The human osteosarcoma tissue microarray was obtained from YBLBIO (Shanghai, China), and its use for scientific research was approved by the Ethics Committee of Hefei DASE Biotechnology Group Co., Ltd (approval No. LLSC‐DASE‐26051807). Databases of human osteosarcoma patients were from GSE152048 and GSE162454 in the NCBI Gene Expression Omnibus (GEO) database repository. Thus, no ethical approval was required for using the hESC lines and patients’ samples for research. Animal experiments in this study were governed by the animal use protocol # UMARE‐011‐2019 and #UMARE‐016‐2020 approved by the University of Macau Animal Research Ethics Sub‐panel.

## Conflicts of Interest

R.‐H.X. is a founder of ImStem Biotechnology, Inc., a stem cell company. The other authors declare no conflicts of interest.

## Supporting information




**Supporting File 1**: advs77726‐sup‐0001‐SuppMat.docx.

**Supporting File 2**: advs77726‐sup‐0002‐TableS5.csv.

**Supporting File 3**: advs77726‐sup‐0003‐TableS6.csv.

## Data Availability

The high‐throughput sequencing data generated in this study have been deposited in the NCBI Sequence Read Archive (SRA) under BioProject accession number PRJNA1509152.
