# Supplementary material for: TP53 Loss Elevates NF‐κB‐IFN‐β‐MHC‐Ia Signaling to Promote NK Cell Resistance in Osteosarcoma
Source: Adv Sci (Weinh). 2026 Sep 11:e77726. Online ahead of print. doi: 10.1002/advs.77726 (PMC13564184; doi:10.1002/advs.77726)
Supplement: Supplementary file 1 [file ADVS-9999-e77726-s001.docx]

**Supplemental Information**

**TP53 Loss Elevates NF-κB-IFNβ-MHC-Ia Signaling to Promote NK Cell Resistance in Osteosarcoma**

Guihui Qin ^1,2, #^, Cheung Kwan Yeung ^1,2, #^, Ye Yi ^1,2^, Dejin Zheng ^1,2^, Miaoman Ye ^1,2^, Chi-Chong Chio^1,2^, Jinjie Wu ^1,2^, Siyi Fu ^1,2^, Chu-Xia Deng ^1,2,3^, Ren-He Xu ^1,2,*^

1 Center of Reproduction, Development & Aging, and Institute of Translational Medicine, Faculty of Medicine, University of Macau, Macao SAR, China.
2 Ministry of Education Frontiers Science Center for Precision Oncology, University of Macau, Taipa, Macau, China.
3 Faculty of Synthetic Biology, Shenzhen University of Advanced Technology, Shenzhen 518107, China.

^*^Corresponding author.

^#^These authors contributed equally to this work.

**Material and methods**

1. **RT-qPCR**

Total RNA was extracted using TRIzol reagent (Thermo Fisher Scientific) according to the manufacturer's instructions. Complementary DNA (cDNA) synthesis was performed using the RT master mix reagent (TaKaRa). Quantitative real-time PCR (qRT-PCR) gene-specific primers (Table S1) was conducted using iTaq Universal SYBR Green Supermix (Bio-Rad Laboratories) with thermal cycling parameters as follows: 95°C for 30 sec, 40 cycles of 95°C for 5 sec, and 60°C for 30 sec. Relative mRNA level was calculated using the 2 delta-delta Ct (2−ΔΔCt) method and GAPDH was measured as an endogenous normalization control.

**Table S1. Sequences of qPCR primers in this study**

| Primer name | Sequences (5’-3’) |
| --- | --- |
| qGAPDH.For | AGGGCTGCTTTTAACTCTGGT |
| qGAPDH.Rev | CCCCACTTGATTTTGGAGGGA |
| qTP53.For | CAGCACATGACGGAGGTTGT |
| qTP53.Rev | TCATCCAAATACTCCACACGC |
| qP21.For | TGTCCGTCAGAACCCATGC |
| qP21.Rev | AAAGTCGAAGTTCCATCGCTC |
| qRB1.For | CTCTCGTCAGGCTTGAGTTTG |
| qRB1.Rev | GACATCTCATCTAGGTCAACTGC |
| qMYC.1For | GGCTCCTGGCAAAAGGTCA |
| qMYC.1Rev | CTGCGTAGTTGTGCTGATGT |
| qTERT.For | TCACGGAGACCACGTTTCAAA |
| qTERT.Rev | TTCAAGTGCTGTCTGATTCCAAT |
| qIRF9.For | GCCCTACAAGGTGTATCAGTTG |
| qIRF9.Rev | TGCTGTCGCTTTGATGGTACT |
| qSTAT1.For | ATCAGGCTCAGTCGGGGAATA |
| qSTAT1.Rev | TGGTCTCGTGTTCTCTGTTCT |
| qSTAT2.For | CTGCTAGGCCGATTAACTACCC |
| qSTAT2.Rev | TCTGATGCAGGCTTTTTGCTG |
| IRF3.forward | AGAGGCTCGTGATGGTCAAG |
| IRF3.reverse | AGGTCCACAGTATTCTCCAGG |
| IRF7.forward | GGCTGGAAAACCAACTTCCG |
| IRF7.reverse | GTTATCCCGCAGCATCACGA |
| CGAS.For | TAACCCTGGCTTTGGAATCAAAA |
| CGAS.Rev | TGGGTACAAGGTAAAATGGCTTT |
| TMEM173.For | AGCATTACAACAACCTGCTACG |
| TMEM173.Rev | GTTGGGGTCAGCCATACTCAG |
| TREX1.forward | TCCCCTTCGGATCTTAACAC |
| TREX1.reverse | CGAAAAAGATGAGGGTCTGC |
| TBK1.For | TGCACCCTGATATGTATGAGAGA |
| TBK1.Rev | AAATGGCAGTGATCCAGTAGC |
| B2M.For | GAGGCTATCCAGCGTACTCCA |
| B2M.Rev | CGGCAGGCATACTCATCTTTT |
| MICA-F | AGGGTTTCTTGCTGAGGTACA |
| MICA-R | GGTCTCTCTGTCCCATGTCTTA |
| MICB-F | TCTTCGTTACAACCTCATGGTG |
| MICB-R | TCCCAGGTCTTAGCTCCCAG |
| ULBP1-F | TAAGTCCAGACCTGAACCACA |
| ULBP1-R | TCCACCACGTCTCTTAGTGTT |
| ULBP2-F | GTGGTGGACATACTTACAGAGC |
| ULBP2-R | CTGCCCATCGAAACTGAACTG |
| ULBP3-F | TCTATGGGTCACCTAGAAGAGC |
| ULBP3-R | TCCACTGGGTGTGAAATCCTC |
| ULBP4-F | CTCTGCAAGTCGAGATGTTTTGT |
| ULBP4-R | TCCATTGGTGGCGAACTGC |
| ULBP5-F | GACAGCTACCAAATAGCGAAGC |
| ULBP5-R | GGTAAGGAGTGTGAGTCGTCT |
| ULBP6-F | TCATCCCTAAGTTCAGACCTGG |
| ULBP6-R | GGACTGACGGGTGTGACTG |
| PVR-F | TGGAGGTGACGCATGTGTC |
| PVR-R | GTTTGGACTCCGAATAGCTGG |
| NECTIN2-F | GGATGTGCGAGTTCAAGTGCT |
| NECTIN2-R | TGGGACCCATCTTAGGGTGG |
| IFNB1-F | TCCCAGGAACTCAATGAAGG |
| IFNB1-R | GTGTCGCAATGGAGTGTTGT |

2. **ELISA**

Cells were seeded in 6-well plates at optimal density and cultured for 48 h under standard conditions. The supernatant (*i.e.*, conditioned media) was collected after centrifugation at 1,000 rpm for 5 min. with fresh medium as a negative control. Human or mouse IFN-β level was assessed using human IFNβ ELISA kit (R&D Systems, DIFNB0) or mouse IFNβ ELISA kit (Huabio, EM0040) following the manufacturer's protocol. Human granzyme B and IFNγ levels were examined using human granzyme B and IFNγ ELISA kits (Huabio, #EH0052 and #EH0039) following the manufacturer's protocols. For Human IFNβ, test samples and recombinant standard curve dilutions (0-1,000 pg mL^-1^) were loaded onto anti-IFNβ pre-coated 96-well microplates and incubated at room temperature with 200 rpm shaking for 2 h. After four washes with a washing buffer, the microplate was incubated with human IFNβ conjugate at room temperature with 200 rpm shaking for 2 h. Following secondary washing, 200 μL Substrate Solution was added for another 30 min incubation. Reactions were terminated with 50 μL Stop Solution and the absorbance at 450 nm was tested within 30 min.

3. **Western blotting**

Total cellular proteins were extracted using RIPA buffer supplemented with protease and phosphatase inhibitors. 20~40 μg total protein was load into SDS-PAGE gel followed by electrophoresis and transfer onto a 0.22 μm PVDF membrane (Bio-Rad Laboratories). PVDF membrane was blocked with 5% notfat milk (Bio-Rad Laboratories) in PBS with 0.1% Tween-20 (PBST) for 1 h at room temperature, followed by overnight incubation at 4°C with the primary antibodies (Table S2) diluted in blocking buffer. After three washes with PBST, associated horseradish peroxidase-conjugated secondary antibodies (1:2,000) were incubated at room temperature for 1 h. Chemiluminescent signals were developed using the ECL Western Blotting Luminol Reagent (Bio-Rad Laboratories) and visualized using the Bio-Rad ChemiDoc MP Imaging System.

4. **Immunofluorescence (IF) staining and tyramide signal amplification (TSA) for tissue array**

Cells were plated into 35 x 10 mm dishes for 24 h. After designated treatments, cells were fixed with 4% paraformaldehyde (PFA) for 15 min. For IF staining of tissue samples, tissues were fixed with 4% PFA, embedded in paraffin, and sectioned into 5 μm thick slices. The tissue slides were deparaffinized and prepared for subsequent use. For cytosolic and nuclear protein detection, cells and slides were treated with 0.1% Triton X-100 for 0.5 h. This step was not needed for detection of membrane proteins. Cells and slides were blocked by 3% BAS buffer for 1 h and then incubated with a primary antibody (Table S2) at 4 ℃ overnight. After 3 washes with PBST and incubation with a secondary antibody, cellular nucleus was stained with 4',6-diamidino-2-phenylindole (DAPI) and images were captured under the fluorescence microscope. Human osteosarcoma tissue array (YP-S-OSCS1602M) was obtained from YBLBIO (Shanghai, China). The p53 and HLA-Ia signals were stained using the tyramide signal amplification (TSA) technique. The p53 and HLA-Ia staining scores were calculated through multiplication of the staining intensity score by the percentage score of positively stained tumor cells. Briefly, staining intensity was scored from 0 to 4 as follows: 0, negative; 1, weak; 2, moderate; 3, moderately strong; and 4, strong staining. The score of positively stained tumor cells was assigned from 0 to 5 as follows: 0, 0%; 1, 1–5%; 2, 6–25%; 3, 26–50%; 4, 51–75%; and 5, >75%.

5. **Flow cytometry**

Cells were digested into single cells. For cytosolic and nuclear protein detection, cells were fixed at -20 ℃ with 90% methanol for 15 min. Fixation was not needed for membrane protein detection. Cells were blocked by 3% BAS buffer for 0.5 h and then incubated with a primary antibody (Table S2) on ice for 1 h. Samples incubated with paired isotype controls were prepared parallelly. After incubation with secondary antibody and washing with PBST, cells were loaded to flow cytometer for detection.

**Supplemental tables**

**Table S2. List of antibodies used in this study**

| Antibody | Brand | Catalog # | Application |
| --- | --- | --- | --- |
| p53 (DO-1) | Santa Cruz | sc-126 | WB (1:200) |
| phospho-p53 (Ser536) | CST | 9286S | WB (1:1000) |
| p21 Waf1/Cip1 | CST | 2946S | WB (1:1000) |
| phospho-STING (Ser366) | CST | 50907 | WB (1:1000) |
| STING | CST | 13647 | WB (1:1000) |
| phospho-TBK1 (Ser172) | CST | 5483 | WB (1:1000) |
| IRF9 | CST | 76684 | WB (1:1000) |
| cGAS | CST | 79978 | WB (1:1000) |
| phospho-IRF3 (Ser396) | CST | 4947 | WB (1:1000) |
| IRF3 | CST | 4302 | WB (1:1000) |
| phospho-STAT1 (Tyr701) | CST | 9167L | WB (1:1000) |
| STAT1 | Santa Cruz | sc-346 | WB (1:200) |
| phospho-IκBα (Ser32) | CST | 2859 | WB (1:1000) |
| IκBα | CST | 4814 | WB (1:1000) |
| phospho-NFκB-p65 (Ser536) | CST | 2859 | WB (1:1000) |
| GAPDH | ABclonal | AC002 | WB (1:10000) |
| β-Actin | BioLegend | 622101 | WB (1:10000) |
| Goat Anti-Rabbit IgG (H+L)-HRP | Bio-Rad | 1706515 | WB (1:2000) |
| Goat Anti-Mouse IgG (H+L)-HRP | Bio-Rad | 1706516 | WB (1:2000) |
| NANOG | Abcam | ab173368 | IF(1:100) Flow (1:100) |
| OCT4 | Invitrogen | PA5-27438 | IF(1:100) Flow (1:100) |
| TRA-1-60 | Abcam | ab16288 | IF(1:100) Flow (1:100) |
| Alkaline Phosphatase/ALPL | R&D | MAB1448SP | IF(1:100) |
| Collagen I | Abcam | ab34710 | IF(1:100) |
| GFP (FITC) | Abcam | ab6662 | IF(1:100) |
| Nkp46 | Invitrogen | PA5-102860 | IF (1:100) |
| CD3ε | CST | 99940 | IF (1:100) |
| HNK-1 | Sigma | C6680-100TST | Flow (1:50) |
| NGFR-p75 | Santa Cruz | sc-56448 | Flow (1:50) |
| HLA-ABC (FITC) | eBioscience | 11-9983-42 | Flow (1:50) |
| HLA-ABC (APC) | eBioscience | 11-9983-43 | Flow (1:50) |
| HLA class I ABC (HLA-Ia) | Proteintech | 66013-1-Ig | WB (1:1000) |
| TAP1 | Proteintech | 1114-1-AP | WB (1:1000) |
| TAP2 | Proteintech | 10161-1-AP | WB (1:1000) |
| MHC class I | Huabio | ET1702-47 | IF (1:200) |
| P63 | Huabio | ET1610-44 | WB (1:1000) |
| P73 | Huabio | ET1609-80 | WB (1:1000) |
| CD3 (FITC) | Invitrogen | 11-0037-42 | Flow (1:50) |
| IFN beta Monoclonal Antibody (A1 (IFNb)), Functional Grade | Invitrogen | 16-9978-81 | 5 μL/ well |

**Table S3. Short tandem repeat results for osteosarcoma cell lines**

| Sample File | Sample Name | Marker | Dye | Allele 1 | Allele 2 |
| --- | --- | --- | --- | --- | --- |
| MG-63_C06.fsa | MG-63 | TH01 | B | 9.3 |  |
|  |  | D21S11 | B | 30 |  |
|  |  | D5S818 | G | 11 | 12 |
|  |  | D13S317 | G | 11 |  |
|  |  | D7S820 | G | 10 |  |
|  |  | D16S539 | G | 11 |  |
|  |  | CSF1PO | G | 10 | 12 |
|  |  | AMEL | Y | X | Y |
|  |  | vWA | Y | 16 | 19 |
|  |  | TPOX | Y | 8 | 11 |
| Saos-2_B06.fsa | Saos-2 | TH01 | B | 6 | 9 |
|  |  | D21S11 | B | 28 | 30 |
|  |  | D5S818 | G | 12 |  |
|  |  | D13S317 | G | 12 | 13 |
|  |  | D7S820 | G | 8 | 10 |
|  |  | D16S539 | G | 12 | 13 |
|  |  | CSF1PO | G | 10 |  |
|  |  | AMEL | Y | X |  |
|  |  | vWA | Y | 18 |  |
|  |  | TPOX | Y | 8 |  |
| U2OS_A04.fsa | U2OS | TH01 | B | 6 | 9.3 |
|  |  | D21S11 | B | 31 |  |
|  |  | D5S818 | G | 11 |  |
|  |  | D13S317 | G | 13 |  |
|  |  | D7S820 | G | 11 | 12 |
|  |  | D16S539 | G | 11 | 12 |
|  |  | CSF1PO | G | 13 |  |
|  |  | AMEL | Y | X |  |
|  |  | vWA | Y | 14 | 18 |
|  |  | TPOX | Y | 11 | 12 |

**Table S4. Off-target screening with whole genome sequenceing**

| chr | location | sequence | mismatch | strand | ref gene | class | observation range | off-target |
| --- | --- | --- | --- | --- | --- | --- | --- | --- |
| 1 | 1:172133811-172133822 | AGCACATGACGG | 0 | + | DNM3 | intron | chr1:172,133,738-172,133,894 | no |
| 1 | 1:41447935-41447946 | AGCACATGACGG | 0 | - | CTPS1 | intron | chr1:41,447,799-41,448,113 | no |
| 1 | 1:15256480-15256491 | AGCACATGACGG | 0 | - | KAZN | exon | chr1:15,256,170-15,256,799 | no |
| 2 | 2:18245804-18245815 | AGCACATGACGG | 0 | + |  | Intergenic | chr2:18,245,525-18,246,139 | no |
| 2 | 2:136577542-136577553 | AGCACATGACGG | 0 | + | LCT | intron | chr2:136,576,932-136,578,162 | no |
| 2 | 2:242101121-242101132 | AGCACATGACGG | 0 | - | PPP1R7 | intron | chr2:242,100,819-242,101,433 | no |
| 2 | 2:220160239-220160250 | AGCACATGACGG | 0 | - | PTPRN | intron | chr2:220,160,168-220,160,320 | no |
| 2 | 2:145188143-145188154 | AGCACATGACGG | 0 | - | ZEB | intron | chr2:145,187,533-145,188,763 | no |
| 2 | 2:127813250-127813261 | AGCACATGACGG | 0 | - | BIN1 | intron | chr2:127,812,948-127,813,562 | no |
| 3 | 3:69987390-69987401 | AGCACATGACGG | 0 | + | MITF | intron | chr3:69,986,393-69,988,397 | no |
| 3 | 3:119385158-119385169 | AGCACATGACGG | 0 | + |  | Intergenic | chr3:119,384,662-119,385,663 | no |
| 4 | 4:129873041-129873052 | AGCACATGACGG | 0 | - | SCLT1 | intron | chr4:129,872,806-129,873,288 | no |
| 4 | 4:10096840-10096851 | AGCACATGACGG | 0 | - | WDR1 | intron | chr4:10,096,606-10,097,088 | no |
| 4 | 4:4277008-4277019 | AGCACATGACGG | 0 | - | LYAR | intron | chr4:4,276,882-4,277,122 | no |
| 4 | 4:2744438-2744449 | AGCACATGACGG | 0 | - | TNIP2 | intron | chr4:2,744,225-2,744,707 | no |
| 5 | 5:41754048-41754059 | AGCACATGACGG | 0 | + | OXCT1 | intron | chr5:41,753,597-41,754,511 | no |
| 5 | 5:132871788-132871799 | AGCACATGACGG | 0 | + | FSTL4 | intron | chr5:132,871,566-132,872,022 | no |
| 6 | 6:8976989-8977000 | AGCACATGACGG | 0 | + |  | Intergenic | chr6:8,976,779-8,977,211 | no |
| 6 | 6:27879740-27879751 | AGCACATGACGG | 0 | + | OR2B2 | exon | chr6:27,872,605-27,886,464 | no |
| 6 | 6:1581568-1581579 | AGCACATGACGG | 0 | - |  | Intergenic | chr6:1,581,141-1,582,006 | no |
| 7 | 7:1629435-1629446 | AGCACATGACGG | 0 | + |  | Intergenic | chr7:1,623,037-1,635,926 | no |
| 7 | 7:49208893-49208904 | AGCACATGACGG | 0 | - |  | Intergenic | chr7:49,202,454-49,215,343 | no |
| 8 | 8:71291594-71291605 | AGCACATGACGG | 0 | + | NCOA2 | intron | chr8:71,290,118-71,293,080 | no |
| 8 | 8:81232345-81232356 | AGCACATGACGG | 0 | - |  | Intergenic | chr8:81,230,870-81,233,832 | no |
| 8 | 8:27981343-27981354 | AGCACATGACGG | 0 | - | ELP3 | intron | chr8:27,981,302-27,981,393 | no |
| 9 | 9:4605498-4605509 | AGCACATGACGG | 0 | + | SPATA6L | intron | chr9:4,605,415-4,605,592 | no |
| 9 | 9:109893217-109893228 | AGCACATGACGG | 0 | + |  | Intergenic | chr9:109,893,045-109,893,401 | no |
| 11 | 11:45185445-45185456 | AGCACATGACGG | 0 | + | PRDM11 | intron | chr11:45,184,768-45,186,133 | no |
| 11 | 11:71965495-71965506 | AGCACATGACGG | 0 | + |  | Intergenic | chr11:71,964,818-71,966,183 | no |
| 11 | 11:69208121-69208132 | AGCACATGACGG | 0 | - |  | Intergenic | chr11:69,207,786-69,208,468 | no |
| 12 | 12:305319-305330 | AGCACATGACGG | 0 | + | SLC6A12 | exon | chr12:304,986-305,662 | no |
| 12 | 12:20124425-20124436 | AGCACATGACGG | 0 | + |  | Intergenic | chr12:20,124,092-20,124,768 | no |
| 12 | 12:59922939-59922950 | AGCACATGACGG | 0 | + |  | Intergenic | chr12:59,922,267-59,923,621 | no |
| 12 | 12:90256961-90256972 | AGCACATGACGG | 0 | + |  | Intergenic | chr12:90,251,546-90,262,387 | no |
| 12 | 12:52828400-52828411 | AGCACATGACGG | 0 | - |  | Intergenic | chr12:52,817,563-52,839,246 | no |
| 14 | 14:22485983-22485994 | AGCACATGACGG | 0 | + |  | Intergenic | chr14:22,485,446-22,486,531 | no |
| 14 | 14:91791349-91791360 | AGCACATGACGG | 0 | - | CCDC88C | intron | chr14:91,790,811-91,791,896 | no |
| 14 | 14:55149702-55149713 | AGCACATGACGG | 0 | - | SMAD4A | intron | chr14:55,149,643-55,149,777 | no |
| 14 | 14:51069041-51069052 | AGCACATGACGG | 0 | - | ATL1 | intron | chr14:51,068,775-51,069,317 | no |
| 15 | 15:42321994-42322005 | AGCACATGACGG | 0 | + | PLA2G4E | intron | chr15:42,321,740-42,322,258 | no |
| 15 | 15:80156313-80156324 | AGCACATGACGG | 0 | + | MTHFS | intron | chr15:80,156,209-80,156,467 | no |
| 15 | 15:81574442-81574453 | AGCACATGACGG | 0 | - | IL16 | intron | chr15:81,574,318-81,574,576 | no |
| 16 | 16:71869634-71869645 | AGCACATGACGG | 0 | - |  | Intergenic | chr16:71,867,811-71,871,469 | no |
| 17 | 17:7129116-7129127 | AGCACATGACGG | 0 | + | DVL2 | 5TUR | chr17:7,128,151-7,131,438 | no |
| 17 | 17:31702332-31702343 | AGCACATGACGG | 0 | + | ASIC2 | intron | chr17:31,702,234-31,702,438 | no |
| 17 | 17:56467395-56467406 | AGCACATGACGG | 0 | + | RNF43 | intron | chr17:56,467,349-56,467,450 | no |
| 17 | 17:78813296-78813307 | AGCACATGACGG | 0 | - | RPTOR | intron | chr17:78,813,096-78,813,506 | no |
| 17 | 17:31049933-31049944 | AGCACATGACGG | 0 | - | MYO1D | intron | chr17:31,049,527-31,050,348 | no |
| 17 | 17:7578419-7578430 | AGCACATGACGG | 0 | - | TP53 | exon | chr17:7,578,218-7,578,628 | on-target |
| 17 | 17:6088991-6089002 | AGCACATGACGG | 0 | - |  | Intergenic | chr17:6,088,788-6,089,198 | no |
| 18 | 18:38924040-38924051 | AGCACATGACGG | 0 | - |  | Intergenic | chr18:38,922,464-38,925,625 | no |
| 19 | 19:46472043-46472054 | AGCACATGACGG | 0 | + | NOVA2 | intron | chr19:46,471,899-46,472,197 | no |
| 20 | 20:54974283-54974294 | AGCACATGACGG | 0 | + | CSTF1 | exon | chr20:54,973,650-54,974,925 | no |
| 20 | 20:42754192-42754203 | AGCACATGACGG | 0 | - | JPH2 | intron | chr20:42,753,543-42,754,818 | no |
| 20 | 20:19218134-19218145 | AGCACATGACGG | 0 | - | SLC24A3 | intron | chr20:19,213,034-19,223,243 | no |
| 20 | 20:3525518-3525529 | AGCACATGACGG | 0 | - | ATRN | intron | chr20:3,525,454-3,525,612 | no |
| 21 | 21:41608485-41608496 | AGCACATGACGG | 0 | + | DSCAM | intron | chr21:41,608,003-41,608,976 | no |
| 21 | 21:45744749-45744760 | AGCACATGACGG | 0 | + | PFKL | exon | chr21:45,744,271-45,745,244 | no |
| 21 | 21:38769495-38769506 | AGCACATGACGG | 0 | - | DYRK1A | intron | chr21:38,769,013-38,769,986 | no |
| X | X:91877779-91877790 | AGCACATGACGG | 0 | - | PCDH11X | exon | chrX:91,877,392-91,878,177 | no |
| Y | Y:17442216-17442227 | AGCACATGACGG | 0 | + |  | Intergenic | chrY:17,439,817-17,444,625 | no |
| Y | Y:5609815-5609826 | AGCACATGACGG | 0 | - | PCDH11Y | exon | chrY:5,607,417-5,612,225 | no |

**Table S5.** **CellChat inferred ligand–receptor interactions in the p53low group (in a separate file)**

**Table S6. CellChat inferred ligand–receptor interactions in the p53high group (in a separate file)**

**Table S7. HLA-Ia genotyping for target cells and match KIR recepters**

| Cell Line | HLA genotype and abundance | Potential matched KIR | Functional Type |
| --- | --- | --- | --- |
| U2OS *TP53^+/+^* | HLA-A (6.77%):  A*02:01:01, A*32:01:01 (Bw4) | KIR3DL1 | Inhibitory |
|  | HLA-B (45.86%):  B*44:345N, B*44:27:01 (Bw4) | KIR3DL1 | Inhibitory |
|  | HLA-C (30.86%):  C*07:04:01, C*07:04:01 | KIR2DL2/KIR2DL3 | Inhibitory |
| U2OS *TP53^-/-^* #1 | HLA-A (10.89%):  A*02:01:01, A*32:01:01 (Bw4) | KIR3DL1 | Inhibitory |
|  | HLA-B (29.06%):  B*44:345N, B*44:27:01 (Bw4) | KIR3DL1 | Inhibitory |
|  | HLA-C (35.54%):  C*07:04:01, C*07:04:01 | KIR2DL2/KIR2DL3 | Inhibitory |
| U2OS *TP53^-/-^* #2 | HLA-A (12.07%):  A*02:01:01, A*32:01:01 (Bw4) | KIR3DL1 | Inhibitory |
|  | HLA-B (28.45%):  B*44:345N, B*44:27:01 (Bw4) | KIR3DL1 | Inhibitory |
|  | HLA-C (39.16%):  C*07:04:01, C*07:04:01 | KIR2DL2/KIR2DL3 | Inhibitory |
| Saos2 *TP53^null^* | HLA-A (12.07%):  A*A*02:01:127, A*A*24:02:01 (Bw4) | KIR3DL1 | Inhibitory |
|  | HLA-B (28.45%):  B*13:07N, B*44:27:01 (Bw4) | KIR3DL1 | Inhibitory |
|  | HLA-C (39.16%):  C*07:04:01, C*07:04:01 | KIR2DL2/KIR2DL3 | Inhibitory |
| MG63 *TP53^null^* | HLA-A (12.07%):  A*01:01:01 (Bw4), A*01:01:01 (Bw4) | KIR3DL1 | Inhibitory |
|  | HLA-B (28.45%):  B*08:01:01 (Bw6), B*08:01:01 (Bw6) | NA | Not classical KIR ligands |
|  | HLA-C (39.16%):  C*07:01:01, C*07:01:01 | KIR2DL2/KIR2DL3 | Inhibitory |
| MSC *TP53^+/+^* | HLA-A (22.01%):  A*02:01:01, A*03:01:01 | KIR3DL2 | Inhibitory |
|  | HLA-B (35.81%):  B*44:27:01, B*44:27:01 | KIR3DL1 | Inhibitory |
|  | HLA-C (29.54%):  C*07:04:01 (Bw4), C*04:456Q | KIR3DL1 | Inhibitory |
| MSC *TP53^-/-^* #1 | HLA-A (18.03%):  A*01:01:01, A*32:01:01 (Bw4) | KIR3DL1 | Inhibitory |
|  | HLA-B (48.29%):  B*14:01:01 (Bw6), B*38:01:01 (Bw4) | KIR3DL1 | Inhibitory |
|  | HLA-C (23.79%):  C*08:02:01, C*12:03:01 | KIR2DL2/KIR2DL3 | Inhibitory |
| MSC *TP53^-/-^* #2 | HLA-A (24.42%):  A*01:01:01, A*32:01:01 (Bw4) | KIR3DL1 | Inhibitory |
|  | HLA-B (43.65%):  B*14:01:01 (Bw6), B*38:01:01 (Bw4) | KIR3DL1 | Inhibitory |
|  | HLA-C (22.58%):  C*08:02:01, C*12:03:01 | KIR2DL2/KIR2DL3 | Inhibitory |

**Table S8. KIR genotyping for primary NK cells.**

| KIR genes | Average Ct value | ΔCt = Ct (KIR) − Ct (KIR3DL3) | 2^-ΔCt | KIR gene status |
| --- | --- | --- | --- | --- |
| *KIR3DL2* | 16.37 | -3.1082 | 8.6232 | present |
| *KIR2DL3* | 18.82 | -0.6592 | 1.5792 | present |
| *KIR3DL1* | 18.83 | -0.6466 | 1.5655 | present |
| *KIR2DP1* | 19.30 | -0.1810 | 1.1337 | present |
| *KIR3DP1-2* | 19.36 | -0.1230 | 1.0890 | present |
| *KIR2DL4* | 19.39 | -0.0863 | 1.0616 | present |
| *KIR2DS4-1* | 19.42 | -0.0599 | 1.0424 | present |
| *KIR3DL3* | 19.48 | -0.0043 | 1.0030 | present |
| *KIR2DL2* | 19.63 | 0.1546 | 0.8984 | present |
| *KIR2DL1* | 19.89 | 0.4134 | 0.7509 | present |
| *KIR2DS4-2* | 21.22 | 1.7365 | 0.3001 | present |
| *KIR2DS3* | 21.22 | 1.7412 | 0.2991 | present |
| *KIR3DS1* | 21.50 | 2.0156 | 0.2473 | present |
| *KIR2DS1* | 22.16 | 2.6829 | 0.1557 | present |
| *KIR2DL5* | 22.32 | 2.8399 | 0.1397 | present |
| *KIR2DS2* | 22.84 | 3.3613 | 0.0973 | present |
| *KIR2DS5* | 23.00 | 3.5208 | 0.0871 | present |
| *KIR3DP1-1* | 25.22 | 5.7354 | 0.0188 | present |

**Supplemental figures**

**Figure S1 (related to Figure 1). Morphology, transcriptomic profiles, and cell-surface HLA-Ia expression of three human osteosarcoma cell lines with differentially *TP53* statuses.** (A) Morphology of osteosarcoma cell lines USO2, Saos2, and MG63. Scale bar, 400 μm. (B) Gene ontology analysis for biological process associated with up-regulated genes in *TP53^-/-^* versus *TP53^+/+^* U2OS cells. (C) Heatmap displaying mRNA expression levels of genes for NK activating and -inhibitory ligands in *TP53^-/-^* versus *TP53^+/+^* U2OS cells. (D) Flow cytometry analysis for HLA-Ia expression in osteosarcoma cells with distinct *TP53* statuses. Representative of three independent experiments (n = 3). (E) ELISA for granzyme B and IFNγ secreted by primary NK cells. n = 3, mean ± SEM, **P* < 0.05, ***P* < 0.01, *****P* < 0.0001 per ordinary two-way ANOVA followed by Šidák’s multiple comparisons test.

**Figure S2 (related to Figures 2 and 3).** (A) Immunoblotting analysis for phosphorylated p53, total p53, and p21 expression in Luc^+^ *TP53^+/+^*, *TP53^-/-^*, and *TP53^OE^* osteosarcoma cell lines. β-actin was tested as an internal control. (B) Flow cytometry analysis for cell-surface expression of HLA-Ia in the osteosarcoma cell lines. Representative of three independent experiments (n = 3). (C) Left: representative images for remaining bioluminescence in NSG mice at different days after intratibial injection. Right: dot plots for remaining bioluminescent signals. Data are shown as mean ± SEM, n = 4. *****P* < 0.0001 per ordinary two-way ANOVA following Tukey’s multiple comparisons test. (D) Immunoblotting of p53, p63, and p73 expression in *TP53^+/+^* and *TP53^-/-^* U2OS clones and Saos2 cells under basal conditions or following X-ray irradiation. GAPDH was tested as an internal control. (E) Representative live-cell fluorescence images showing CD8^+^ T cell killing of GFP-labeled osteosarcoma cells. Data are shown as mean ± SEM, n = 4, *****P* < 0.0001 per ordinary two-way ANOVA with Dunnett’s multiple comparisons test. (F) Representative images of H&E staining on sections of the *Trp53^+/+^* and *Trp53^-/-^* K7M2 tumors in both intramuscular and intratibial injection model. Scale bar, 100 μm.

**Figure S3 (related to Figure 4). Single-cell dynamics and *TP53* network remodeling in osteosarcoma tumorigenesis.** (A) Stacked bar plot showing cell type proportions across 17 primary osteosarcoma specimens. n = 11 from GSE152048 marked as BC, n = 6 from GSE162454 marked as OS. (B) CytoTRACE 2 potency scores mapped onto UMAP coordinates with emerging gradient from differentiated (blue) to pluripotent (red) states. Potency score is a continuous value ranging from 0 (differentiated), 0.2 (unipotent), 0.4 (oligopotent), 0.6 (multipotent), 0.8 (pluripotent) to 1 (totipotent). (C) Global distribution of of spliced/unspliced/ambiguous mRNA percentage (left panel). Average of spliced/unspliced/ambiguous mRNA percentage of MSCs, osteoblasts, and proliferating osteoblasts (right panel). (D) CNV scores mapped onto UMAP plot. (E) Violin plot of p53 functional scores based on ssGSEA scores of the hallmark p53 pathway. (F) Circle plot showing interaction weights among different cell populations in p53^low^ and p53^high^ tumor samples.

**Figure S4 (related to Figure 5). Off-target analysis of *TP53*-specific CRISPR-Cas9 KO.** (A) Computational prediction of top 6 target sites for the *TP53*-specific sgRNA sequences, including one on-target site and 5 off-target sites, based on Cas-OFFinder (http://www.rgenome.net/cas-offinder/). (B) Sanger sequencing traces of the two *TP53^-/-^* clones (#1-2), in comparison with the WT sequence, at predicted loci.

**Figure S5 (related to Figure 5). *TP53* KO preserves pluripotency in hESCs.** (A) Karyotyping for *TP53^+/+^* hESCs and two *TP53^-/-^* isogenic clones. (B-C) Immunofluorescence and flow cytometry analyses for pluripotency markers NANOG, OCT4, and TRA-1-60 in the *TP53^+/+^* and *TP53^-/-^* hESCs, Scale bar, 50 μm. (D) Teratomas formed by the *TP53^+/+^* and *TP53^-/-^* hESCs. scale bar, 1.0 cm. (E) H&E staining of sections from the teratomas in D. Representative cells from the three germ layers were marked with arrows in different colors as designated below.

**Figure S6 (related to Figure 5). HESC differentiation into MSCs via an intermediate stage of NCCs.** (A) Schematic of the strategy for *RB1* KO and genotyping validation for two *RB1^-/-^* hESC clones #1 and #2. (B) Schematic graph for hESC differentiation into MSCs via an intermediate stage of NCCs. Scale bar, 400 μm. Representative images for the three cell types are shown below. (C-D) Immunofluorescence (C) and flow cytometry (D) analyses for HNK-1 and NGFR-p75 in NCCs differentiated from the *TP53^+/+^* hESCs and two *TP53^-/-^* isogenic clones. Scale bar, 50 μm. (E) Representative images for osteocytes, adipocytes, and chondrocytes that differentiated from the *TP53^+/+^*, *TP53**^-/-^*, and *TP53^-/-^/RB1^-/-^* MSCs. (F) Average Ct value of *TERT* in the *TP53^-/-^*/*RB1^-/-^* MSCs (control) and the *TP53^-/-^*/*RB1^-/-^*/*TERT^OE^* MSCs (upper panel), and relative *c-MYC* mRNA level in *TP53^-/-^*/*RB1^-/-^*/*TERT^OE^* MSCs (control) and *TP53^-/-^/RB1^-/-^/TERT^OE^/c-MYC^OE^* MSCs (lower pane). Scale bar: 100 μm. n = 3, mean ± SD. ***P* < 0.01, *****P* < 0.001 per unpaired t test. (G) Schematic for three assays to evaluate the osteosarcoma-modeling potency. In all the quantification graphs, error bars represent mean with standard error of mean (SEM). (H) Immunoblotting for p53, p63 and p73 expression in *TP53^+/+^* and *TP53^-/-^* ESC clones and MSCs under basal conditions or following X-ray irradiation. GAPDH was tested as an internal control.

**Figure S7 (related to Figure 6). Detection of IFNβ-triggered expression of NK-regulatory ligands.** (A-B) Relative mRNA level of genes for NK activating (A) and -inhibitory (B) ligands in *TP53^+/+^* and *TP53^-/-^* MSCs. Data are representative of three independent experiments as mean ± SEM. n = 3. Ns, non-significance. **P* < 0.05, ***P* < 0.01, ****P* < 0.001, *****P* < 0.001 per ordinary one-way ANOVA test. (C) ELISA analysis for IFNβ secreted by the MSCs in the presence of human primary NK cells. Data are representative of three independent experiments as mean ± SEM. n = 3. ***P* < 0.01, *****P* < 0.001 per ordinary one-way ANOVA test. (D) Flow cytometry analysis of cell-surface expression of HLA-Ia in MSCs following treatment with IFNβ at various concentrations for 24 h. (E) Immunoblotting for p-STAT1, total STAT1, IRF9, TAP1, and TAP2 expression in MSCs. GAPDH was tested as an internal control. (F) Immunoblotting for p-STAT1, total STAT1, HLA-Ia, TAP1, and TAP2 expression in MSCs treated with IFN-β +/- neutralizing IFN-β antibody. β-actin was tested as an internal control. (G) Flow cytometry analysis of cell-surface expression of HLA-Ia. Representative of three independent experiments. n = 3. (H) Immunoblotting for p-STAT1, total STAT1, HLA-Ia, p- NF-κB, and NF-κB expression in MSCs. β-actin was tested as an internal control. In all the quantification graphs, error bars represent mean with SEM.

**Figure S8 (related to Figure 7). Detection of effectors in the NF-κB-IFNβ-HLA-Ia signaling in *TP53^+/+^* and *TP53^-/-^* MSCs.** (A) Relative mRNA level of designated genes in MSCs. Data are representative of three independent experiments as mean ± SEM. n = 3. ns, non-significance. **P* < 0.05, ***P* < 0.01, ****P* < 0.001, *****P* < 0.001 per ordinary one-way ANOVA test. (B) Immunoblotting analysis of designated proteins in MSCs. β-actin was tested as an internal control. (C) . (D) Relative mRNA level for *IFNB1* in MSCs in the presence of human primary NK cells with or without treatment of the NF-κB inhibitor QNZ at 10 nM for 24 h. Data are representative of three independent experiments as mean ± SEM. n = 3, ns: non-significance, **P* < 0.05, ***P* < 0.01 per unpaired t test with Welch correction. (E) Immunoblotting analysis of designated proteins in osteosarcoma cells. GAPDH was tested as an internal control. (F) ELISA analysis of IFNβ secreted by the osteosarcoma cells. Data are representative of three independent experiments as mean ± SEM. n = 3, ns, non-significant. **P* < 0.05, ***P* < 0.01 per ordinary one-way ANOVA test. In all the quantification graphs, error bars represent mean with SEM.

**Figure S9. Binary alignment map (BAM) analysis of the *TP53* locus in specimens in the osteosarcoma dataset.** (A) Representative BAM data for wide-type *TP53*. (B) Representative BAM data for the loss of *TP53*.
